# Supplementary material for: Isolation of diborenes and their 90°-twisted diradical congeners
Source: Nat Commun. 2018 Mar 22;9:1197. doi: 10.1038/s41467-018-02998-3 (PMC5864745; doi:10.1038/s41467-018-02998-3)
Supplement: Supplementary file 1 — Supplementary Information(PDF 4825 kb) [file 41467_2018_2998_MOESM1_ESM.pdf]

Isolation of Diborenes and their 90º-Twisted Diradical Congeners

Böhnke et al.

Supplementary figures

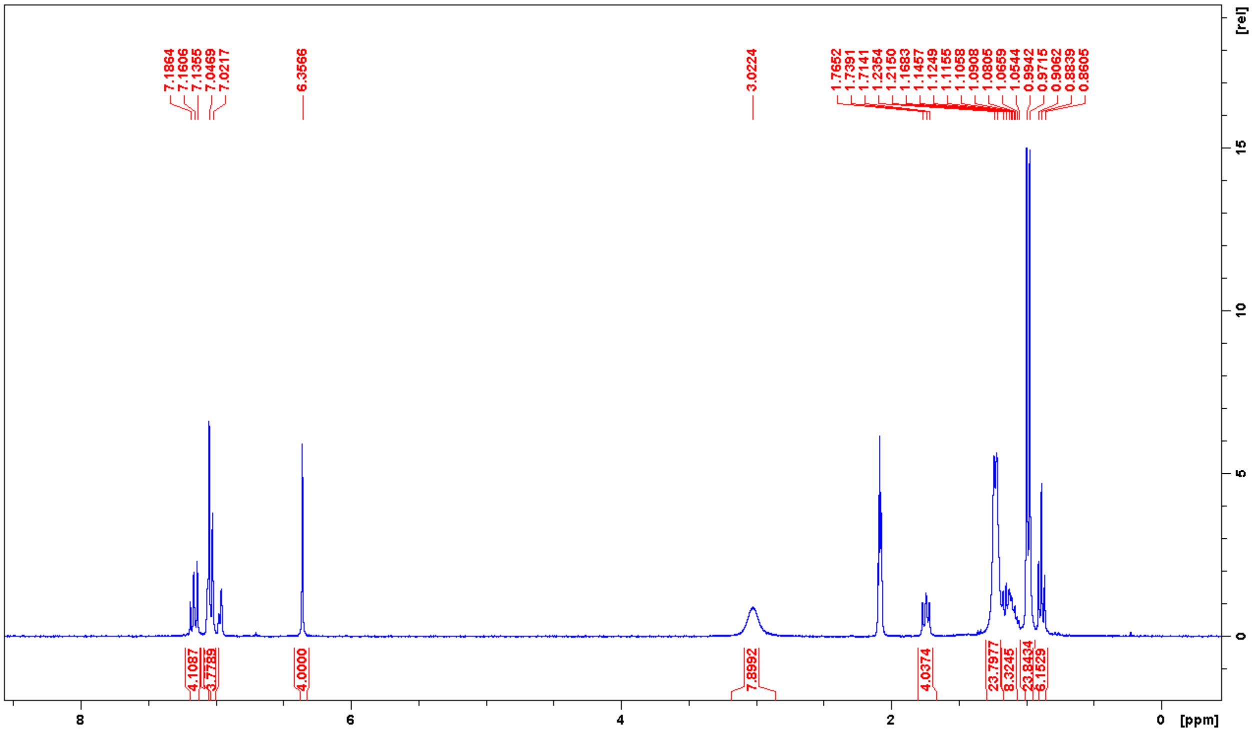

Supplementary Figure 1. ^{1}H NMR spectrum of $B_2(IDip)_2(SBu)_2$ (**1a**) in toluene-d_8 at 80 °C.

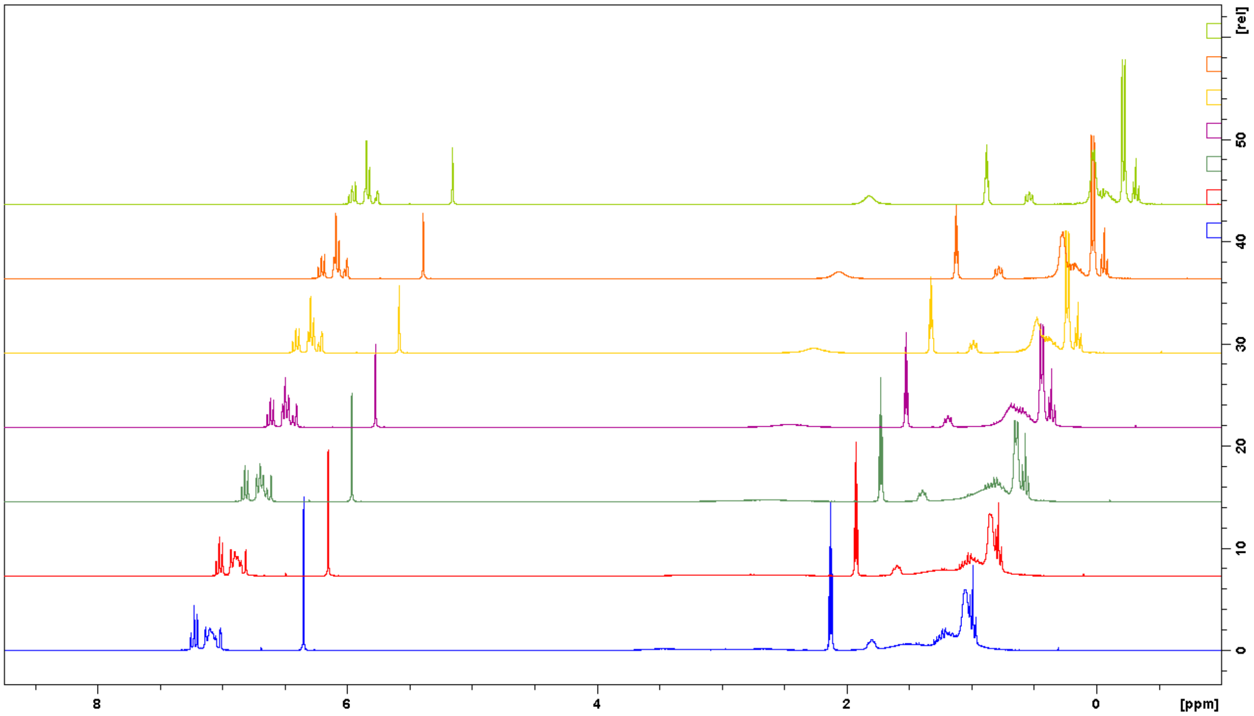

Supplementary Figure 2. ^{1}H NMR spectrum of $B_2(IDip)_2(SBu)_2$ (**1a**) in toluene-d_8 at 25 °C (blue), 30 °C (red), 40 °C (dark green), 50 °C (purple), 60 °C (yellow), 70 °C (orange), 80 °C (bright green).

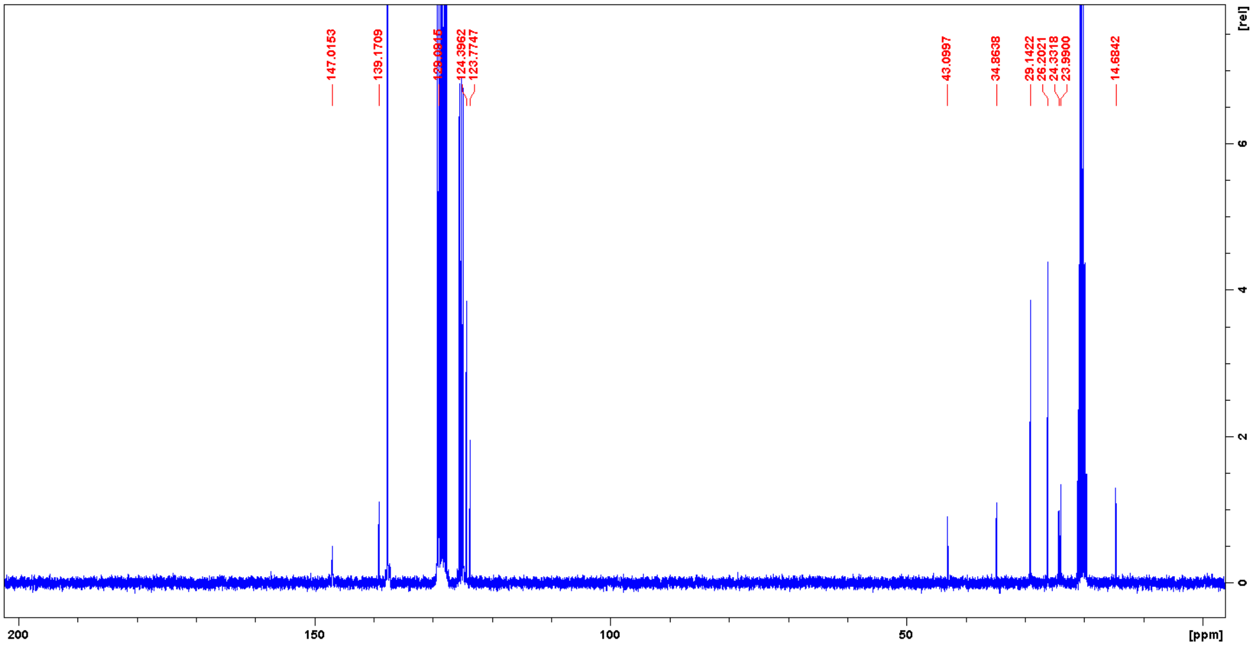

Supplementary Figure 3. $^{13}C\{^{1}H\}$ NMR spectrum of $B_2(IDip)_2(SBu)_2$ (**1a**) in toluene-d_8 at 80 °C.

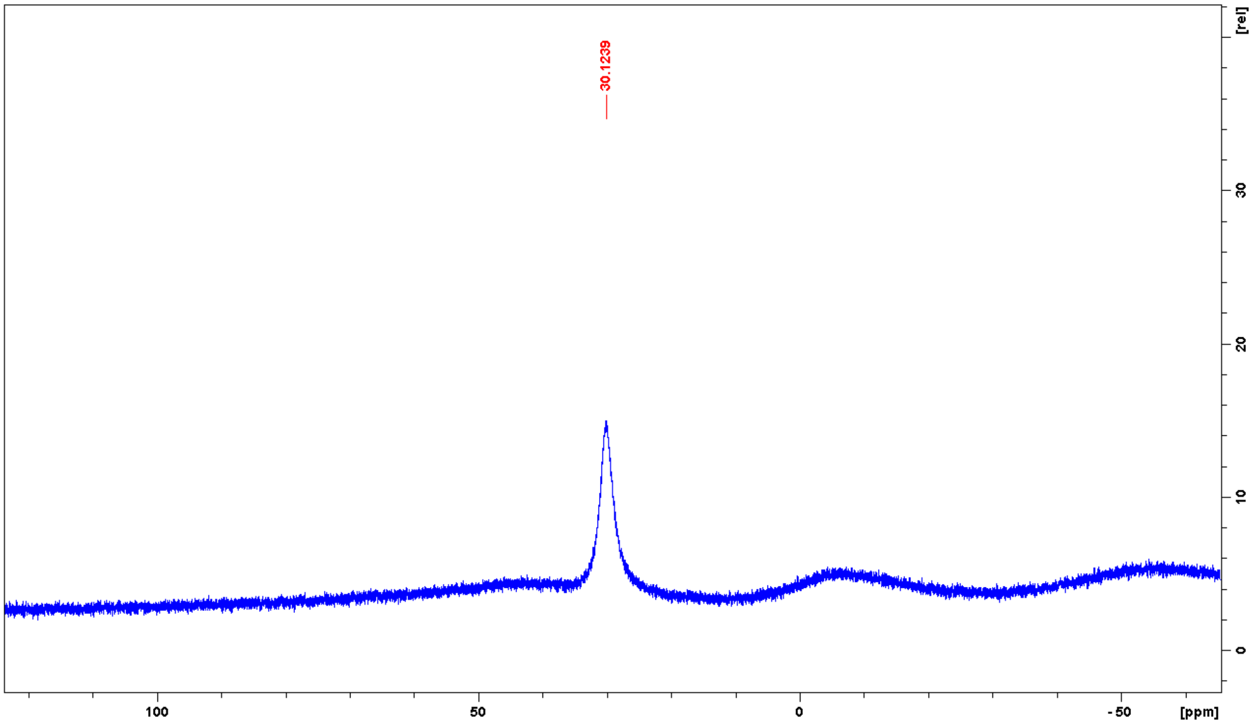

Supplementary Figure 4. ^{11}B NMR spectrum of $B_2(IDip)_2(SBu)_2$ (**1a**) in toluene-d_8 at 80 °C.

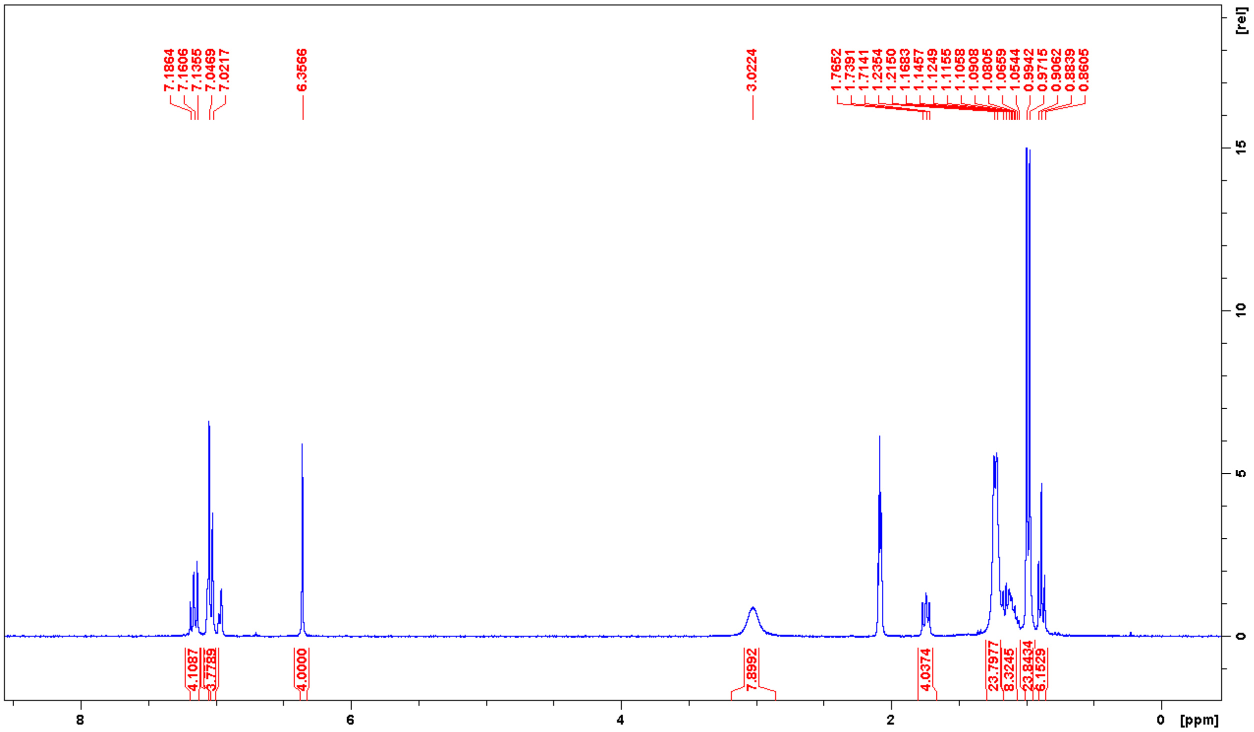

Supplementary Figure 5. ^{1}H NMR spectrum of $B_2IDip_2S_2Ph_2$ (**1b**) in C_6D_6.

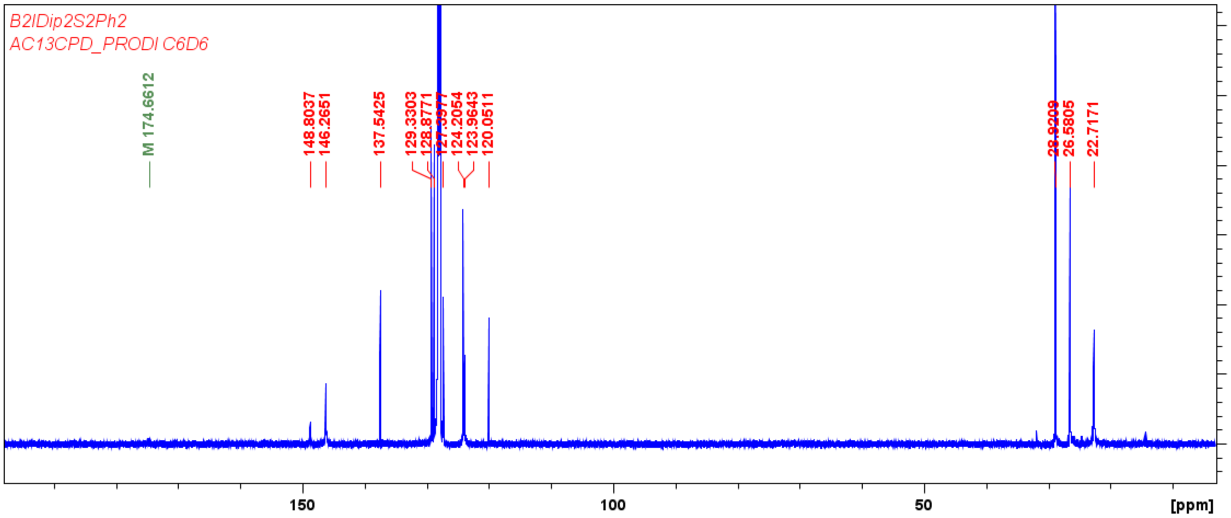

Supplementary Figure 6. $^{13}C\{^{1}H\}$ NMR spectrum of $B_2IDip_2S_2Ph_2$ (**1b**) in C_6D_6.

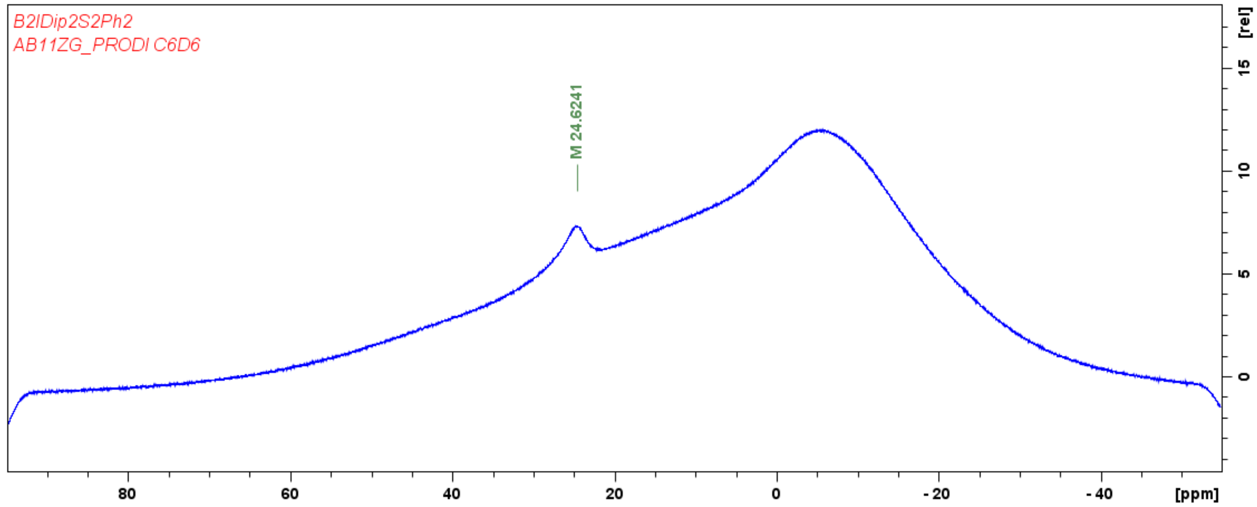

Supplementary Figure 7. ^{11}B NMR spectrum of $B_2IDip_2S_2Ph_2$ (**1b**) in C_6D_6.

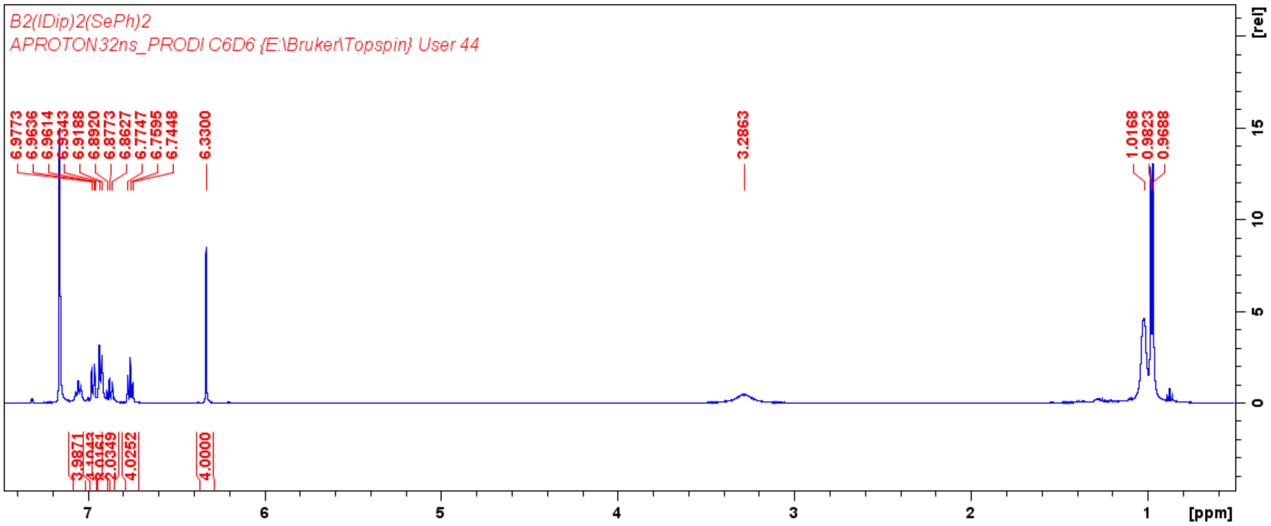

Supplementary Figure 8. ^{1}H NMR spectrum of $B_2IDip_2Se_2Ph_2$ (**1c**) in C_6D_6.

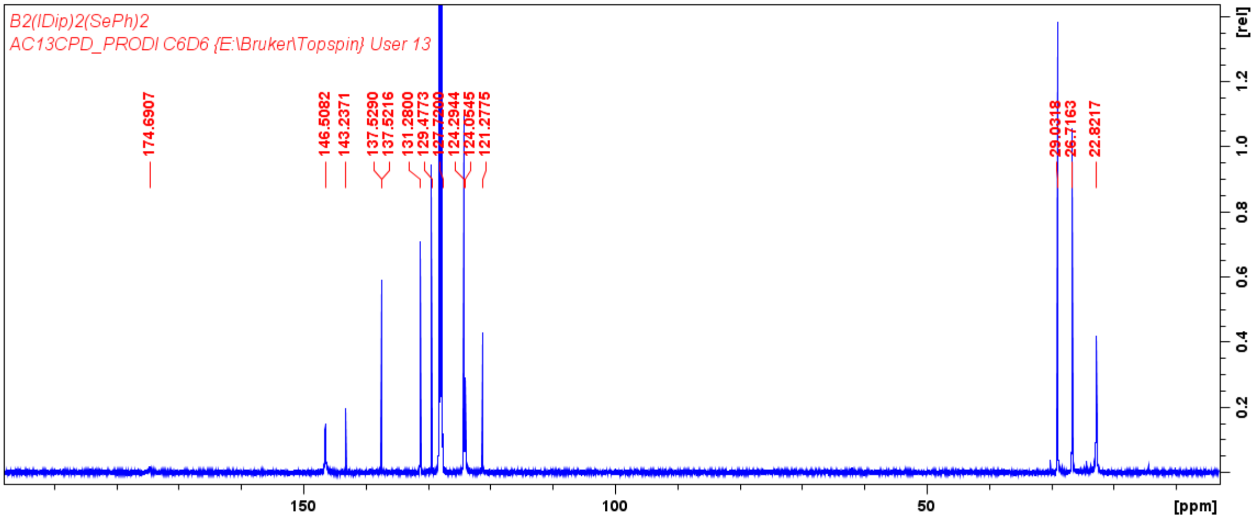

Supplementary Figure 9. $^{13}C\{^1H\}$ NMR spectrum of $B_2IDip_2Se_2Ph_2$ (**1c**) in C_6D_6.

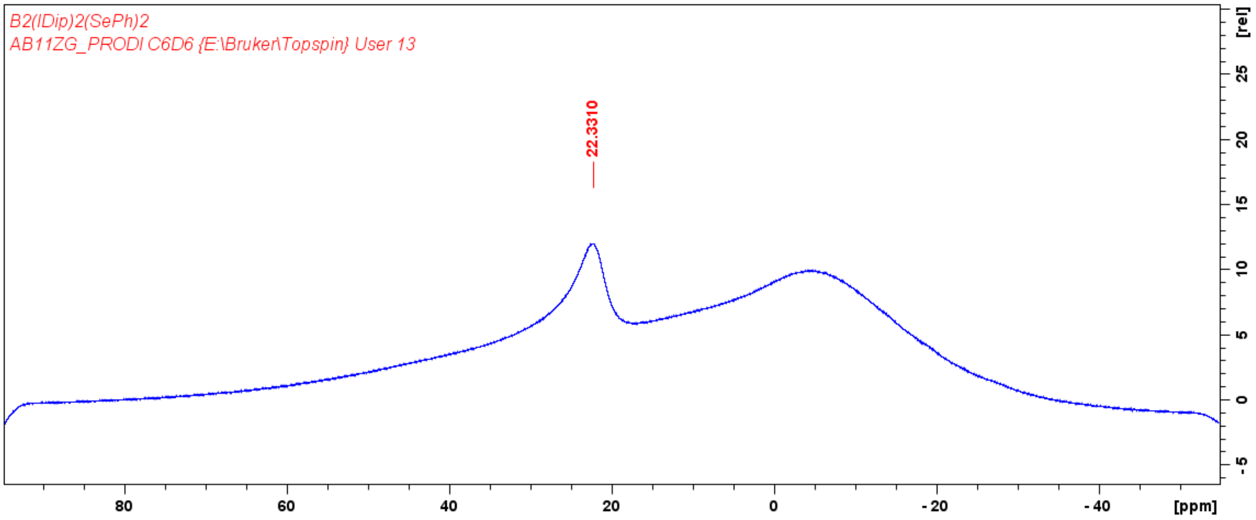

Supplementary Figure 10. ^{11}B NMR spectrum of $B_2IDip_2Se_2Ph_2$ (**1c**) in C_6D_6.

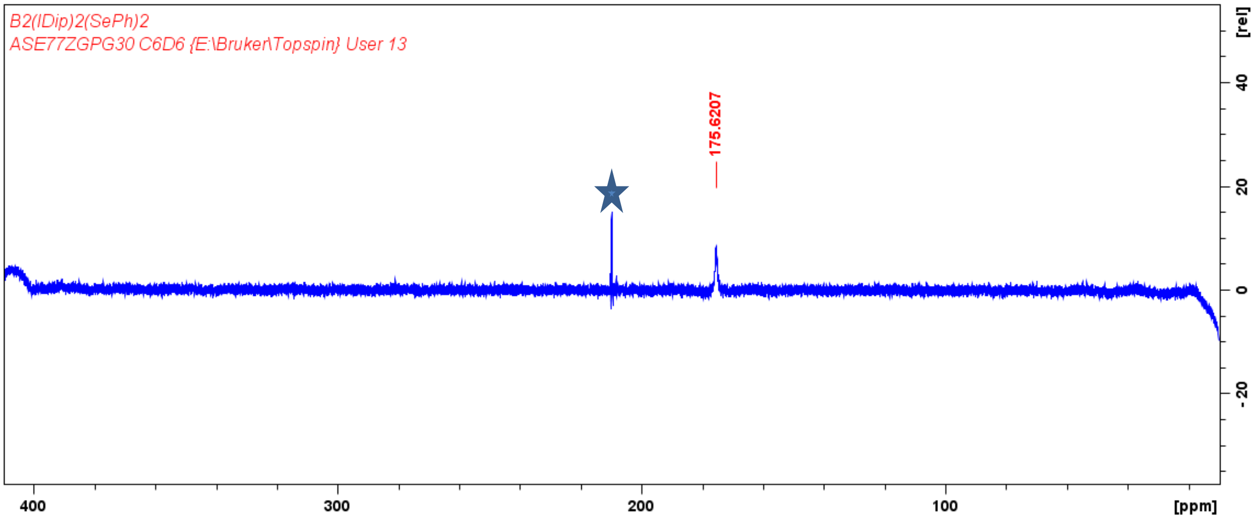

Supplementary Figure 11. ^{77}Se NMR spectrum of $B_2IDip_2Se_2Ph_2$ (**1c**) in C_6D_6 (* artifact of NMR measurement).

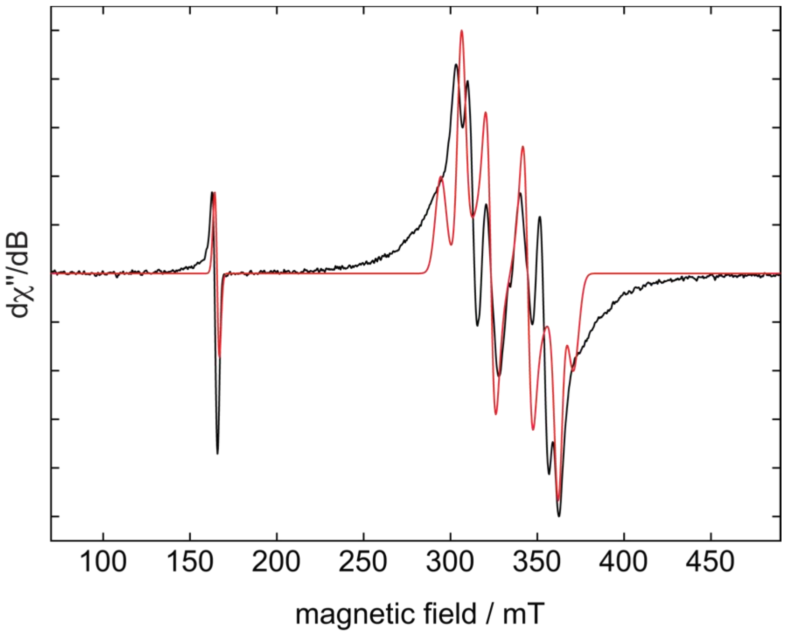

Supplementary Figure 12. Experimental (black) and simulated (red) continuous-wave (CW) X-band EPR spectra of 0.4 mM [$B_2(CAAC)_2(SBu)_2$] (**2a**) in THF at 20 K. The tailing of the outer-most signals in the $|\Delta m_S| = 1$ region was observed even at very low concentrations of 0.1 mM and when using different solvents or solvent mixtures. The simulation parameters for the $S = 1$ state are $|D/hc| = 0.036$ cm^{-1}, $|E/hc| = 0.0054$ cm^{-1}, $g_1 = 2.005$, $g_2 = 2.007$, and $g_3 = 2.009$.

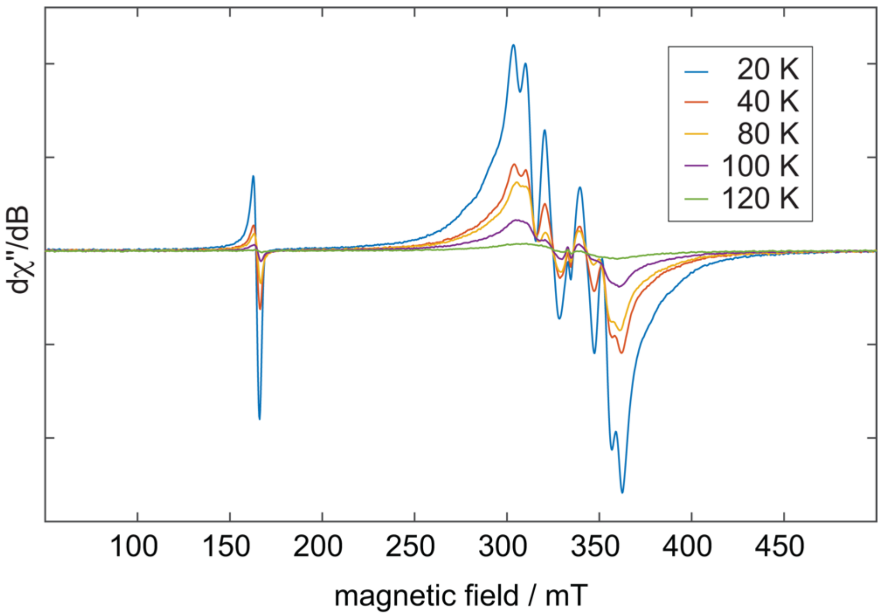

Supplementary Figure 13. Temperature-dependent EPR spectra of 1 mM $[B_2(CAAC)_2(SBu)_2]$ (**2a**) in 2-Me-THF. Experimental parameters: temperature = 20–120 K; microwave frequency = 9.38 GHz; microwave power = 0.1 mW; modulation amplitude = 2 G; conversion time = 60 ms; modulation frequency = 100 kHz.

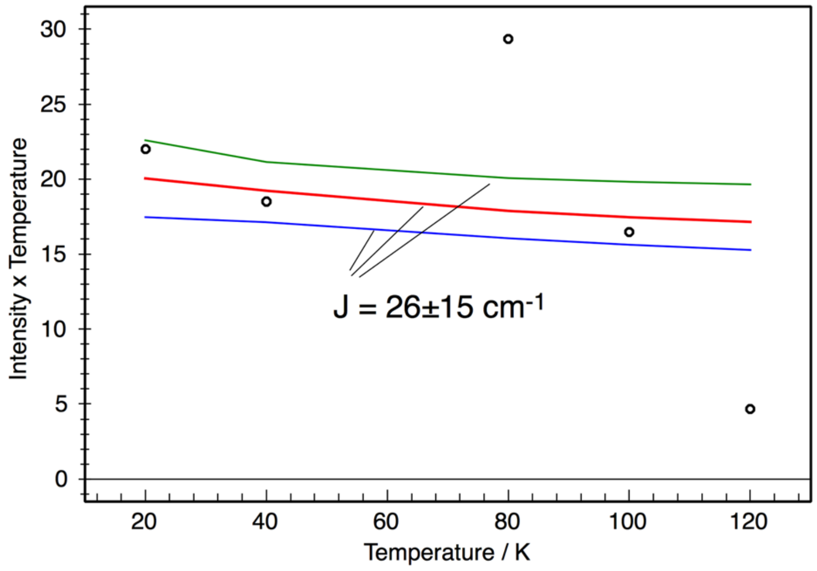

Supplementary Figure 14. Plot of the product of the EPR intensity and the temperature vs. temperature in the 20–120 K temperature range for diradical **2a**. The empty circles correspond to the experimental data, the solid lines to fits (*J* = exchange coupling).

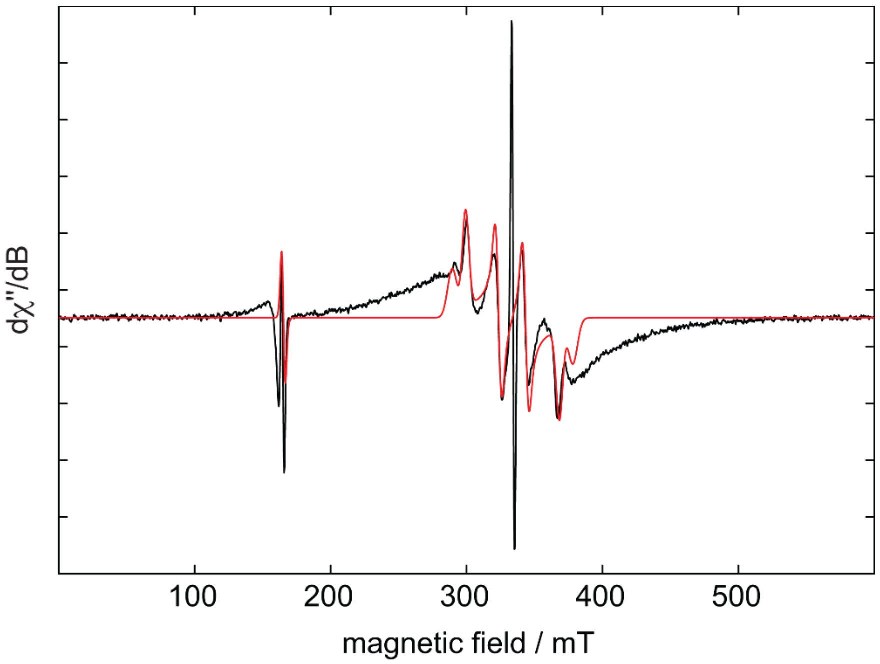

Supplementary Figure 15. Experimental (black) and simulated (red) continuous-wave (CW) X-band EPR spectra of 0.2 mM $[B_2(CAAC)_2(SPh)_2]$ (**2b**) in THF at 20 K. Hyperfine couplings to the boron and nitrogen nuclei are broad and unresolved in the $|\Delta m_S| = 1$ region and have thus not been included in the simulation. The simulation parameters for the $S = 1$ state are $|D/hc| = 0.042$ cm^{-1}, $|E/hc| = 0.0077$ cm^{-1}, $g_1 = 2.005$, $g_2 = 2.007$, and $g_3 = 2.009$. The center line corresponds to an unknown monoradical byproduct.

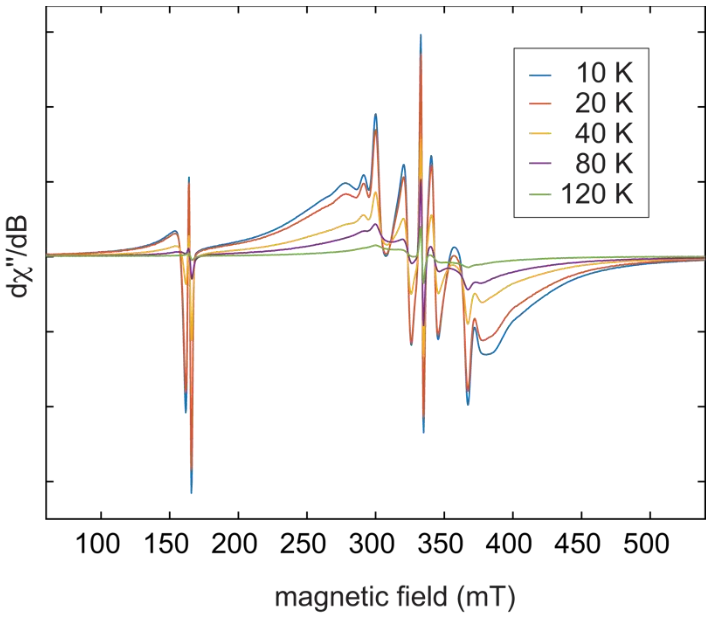

Supplementary Figure 16. Temperature-dependent EPR spectra of 1 mM [$B_2(CAAC)_2(SPh)_2$] (**2b**) in 2-Me-THF. Experimental parameters: temperature = 20−120 K; microwave frequency = 9.38 GHz; microwave power = 1 mW; modulation amplitude = 2 G; conversion time = 120 ms; modulation frequency = 100 kHz.

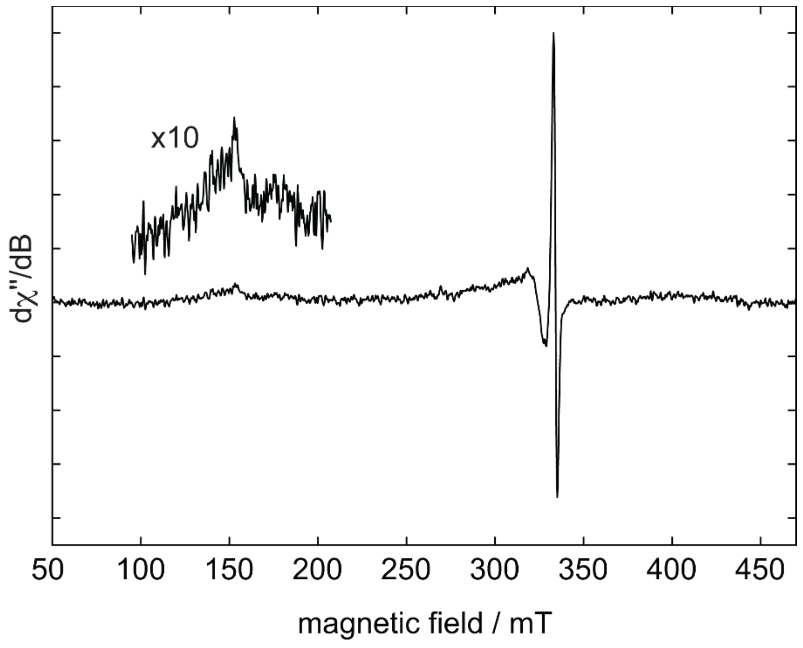

Supplementary Figure 17. EPR spectrum of 1 mM [$B_2(CAAC)_2(SePh)_2$] (**2c**) in 2-Me-THF at 20 K. Experimental parameters: microwave frequency = 9.38 GHz; microwave power = 0.4 mW; modulation amplitude = 2 G; conversion time = 80 ms; modulation frequency = 100 kHz. The center line corresponds to an S = 1/2 byproduct.

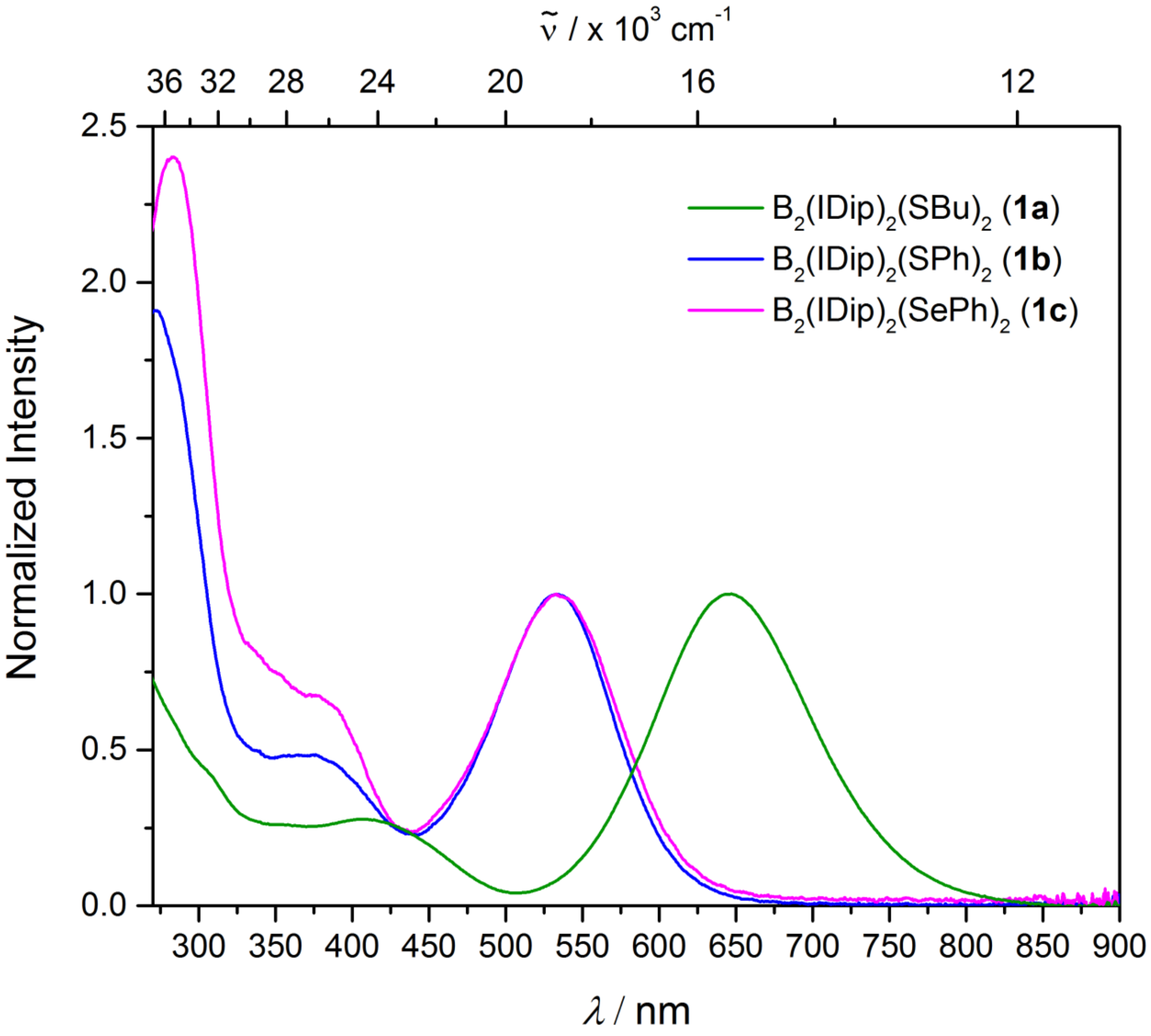

Supplementary Figure 18. Overlay of UV-vis spectra of compounds $B_2(IDip)_2(SBu)_2$ (**1a**, green), $B_2(IDip)_2(SPh)_2$ (**1b**, blue) and $B_2(IDip)_2(SePh)_2$ (**1c**, pink) in pentane. $B_2(IDip)_2(SBu)_2$: c = 4.50 x 10^{-5} M; $B_2(IDip)_2(SPh)_2$: c = 1.53 x 10^{-5} M; $B_2(IDip)_2(SePh)_2$: c = 9.72 x 10^{-6} M.

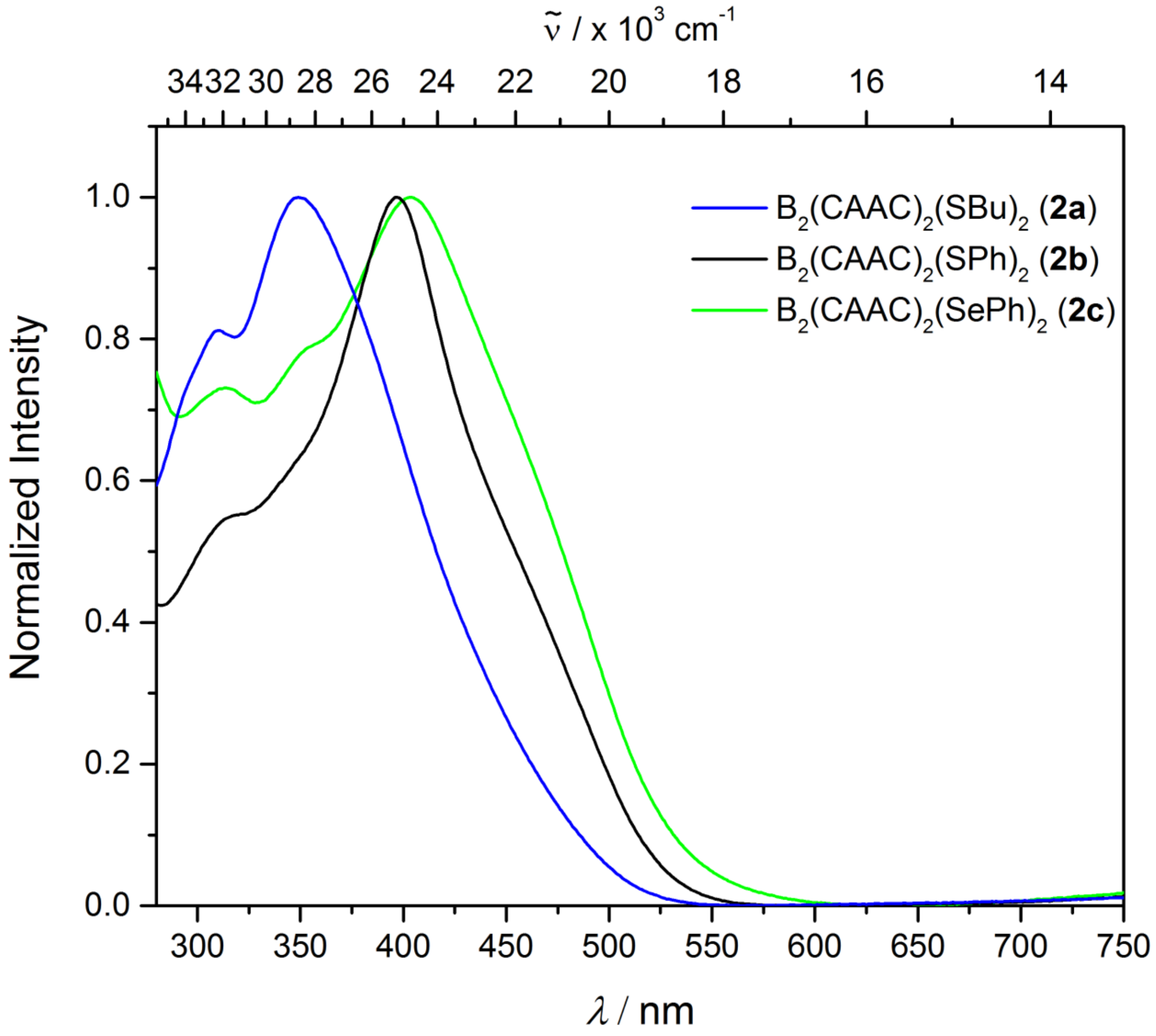

Supplementary Figure 19. Overlay of UV-vis spectra of compounds $B_2(CAAC)_2(SBu)_2$ (**2a**, blue), $B_2(CAAC)_2(SPh)_2$ (**2b**, black) and $B_2(CAAC)_2(SePh)_2$ (**2c**, green) in THF. $B_2(CAAC)_2(SBu)_2$: c = 7.26 x 10^{-5} M; $B_2(CAAC)_2(SPh)_2$: c = 6.91 x 10^{-5} M; $B_2(CAAC)_2(SePh)_2$: c = 6.63 x 10^{-5} M.

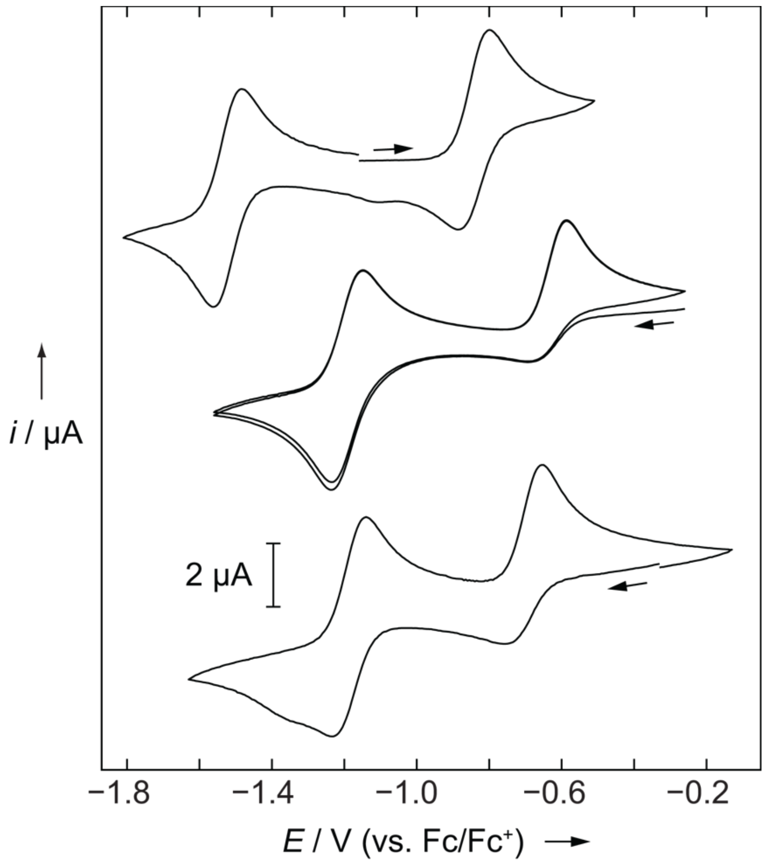

Supplementary Figure 20. Cyclic voltammograms of $B_2(IDip)_2(SBu)_2$ (**1a**, top), $B_2(IDip)_2(SPh)_2$ (**1b**, middle), and $B_2(IDip)_2(SePh)_2$ (**1c**, bottom) in THF/0.1 M $[Bu_4N][PF_6]$ measured at 250 mV s^{-1}. Formal potentials: **1a**: $E_{1/2} = -1.52$ V, $E_{pa} = -0.84$ V; **1b**: $E_{1/2} = -1.18$ V, $E_{pa} = -0.58$ V; **1c**: $E_{1/2} = -1.18$ V, $E_{pa} = -0.65$ V (relative to the Fc/Fc^+ couple).

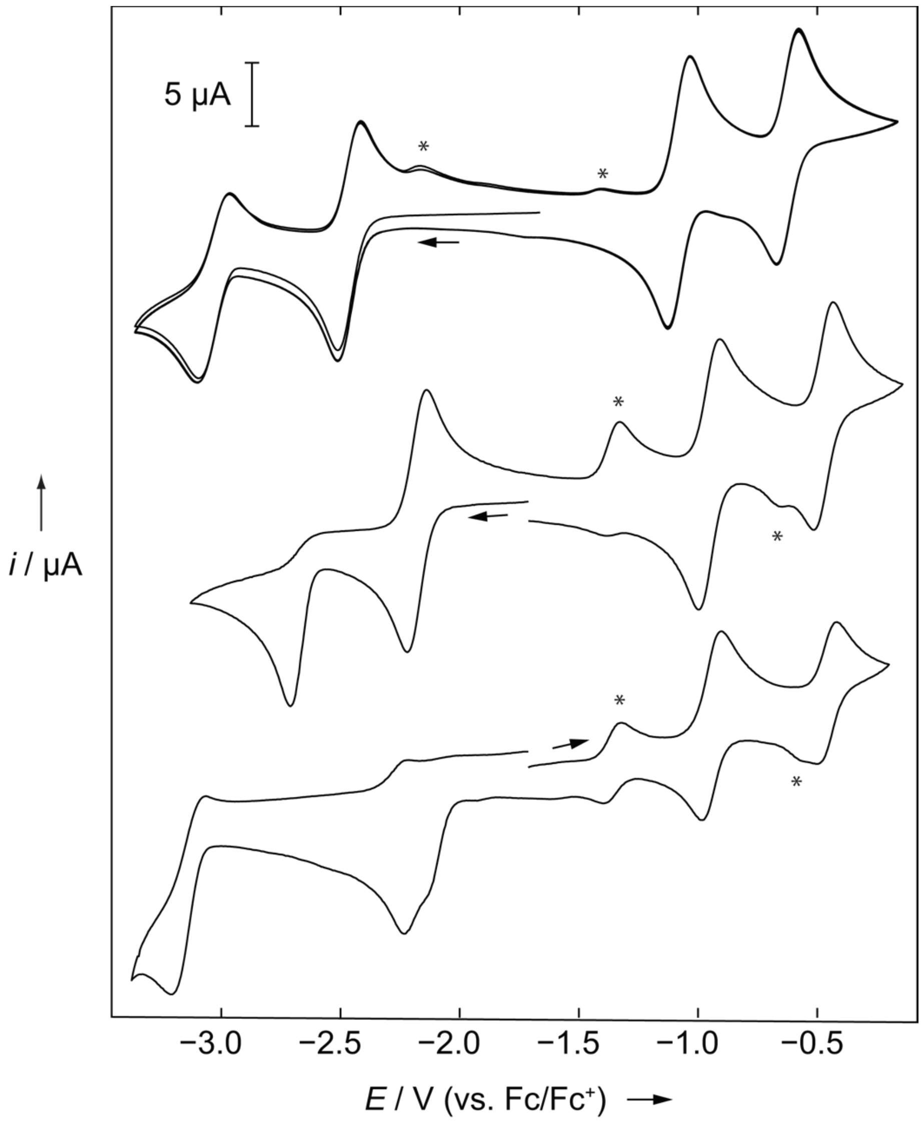

Supplementary Figure 21. Cyclic voltammograms of $B_2(CAAC)_2(SBu)_2$ (**2a**, top), $B_2(CAAC)_2(SPh)_2$ (**2b**, middle), and $B_2(CAAC)_2(SePh)_2$ (**2c**, bottom) in THF/0.1 M $[Bu_4N][PF_6]$ measured at 250 mV s^{-1}. Formal potentials: **2a**: $E_{1/2}$ = −0.62 V, $E_{1/2}$ = −1.07 V; $E_{1/2}$ = −2.45 V, $E_{1/2}$ = −3.01 V; **2b**: $E_{1/2}$ = −0.49 V, $E_{1/2}$ = −0.96 V; $E_{1/2}$ = −2.17 V, E_{pc} = −2.70 V; **2c**: $E_{1/2}$ = −1.33 V, $E_{1/2}$ = −0.91 V, $E_{1/2}$ = −0.43 V; E_{pc} = −2.21 V, E_{pc} = −3.19 V (relative to the Fc/Fc^+ couple). Unknown redox events are marked with an asterisk (*).

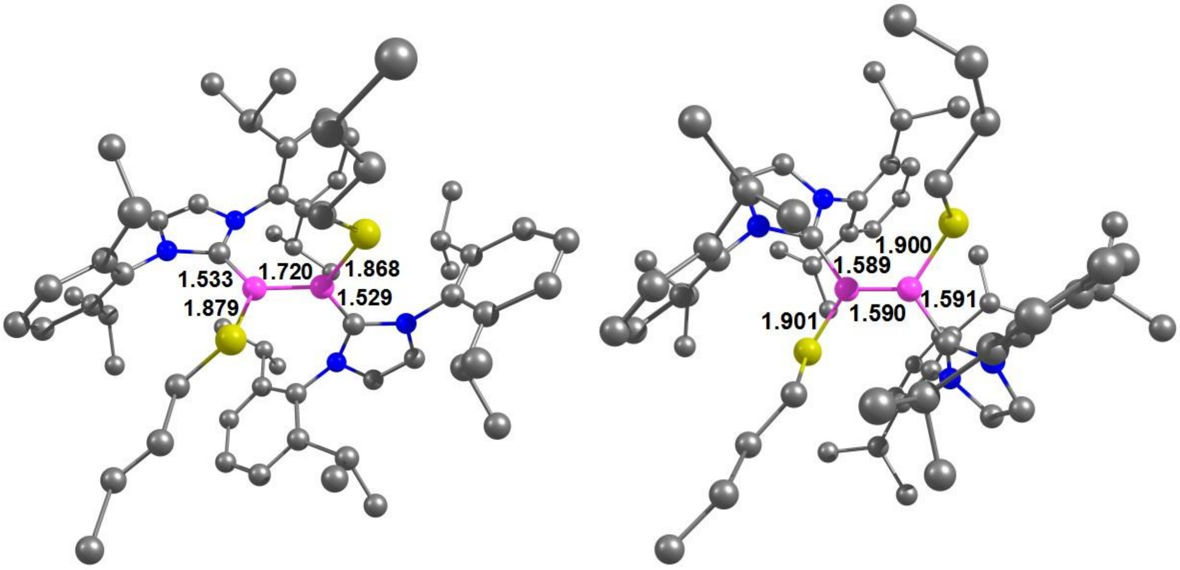

0.0 T1

-16.7 S0

Supplementary Figure 22. Optimized geometries of triplet (left) and singlet (right) states of **1a** at the M05-2X/def2-SVP level. The energies are in kcal mol^{-1} and bond lengths are in Å.

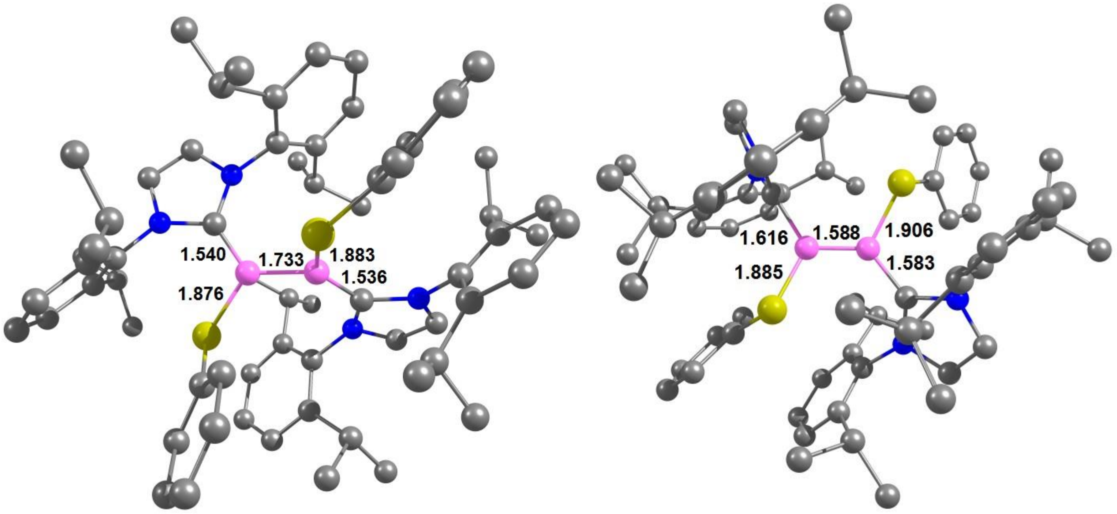

0.0 T1

-24.4 S0

Supplementary Figure 23. Optimized geometries of triplet (left) and singlet (right) states of **1b** at the M05-2X/def2-SVP level. The energies are in kcal mol^{-1} and bond lengths are in Å.

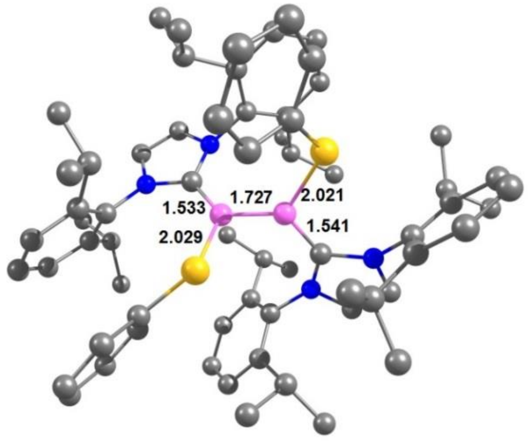

0.0

T1

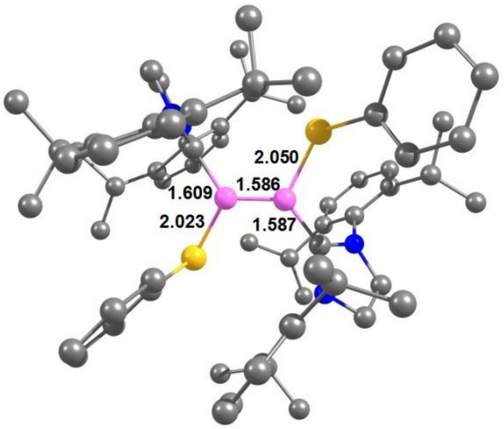

-26.3

S0

Supplementary Figure 24. Optimized geometries of triplet (left) and singlet (right) states of **1c** at the M05-2X/def2-SVP level. The energies are in kcal mol^{-1} and bond lengths are in Å.

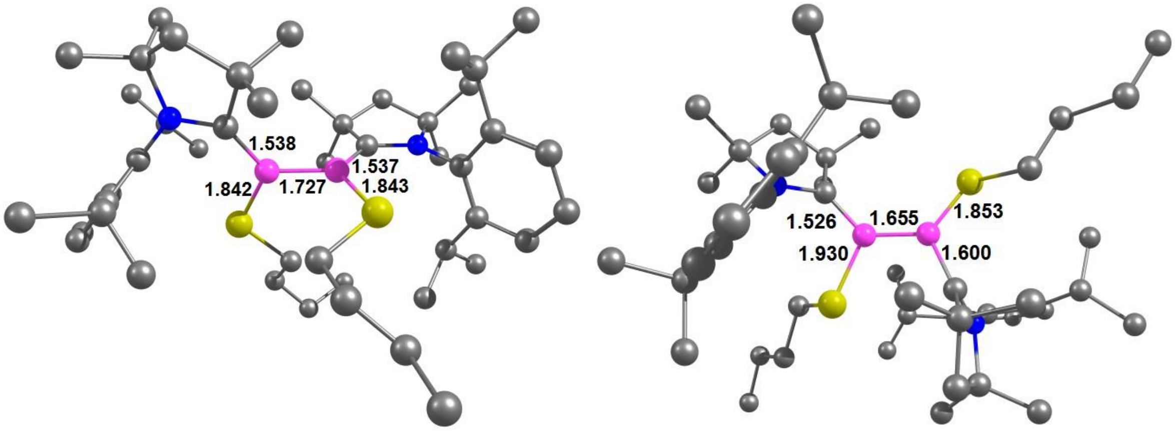

0.0 T1

33.2 S0

Supplementary Figure 25. Optimized geometries of triplet (left) and singlet (right) states of **2a** at the M05-2X/def2-SVP level. The energies are in kcal mol^{-1} and bond lengths are in Å.

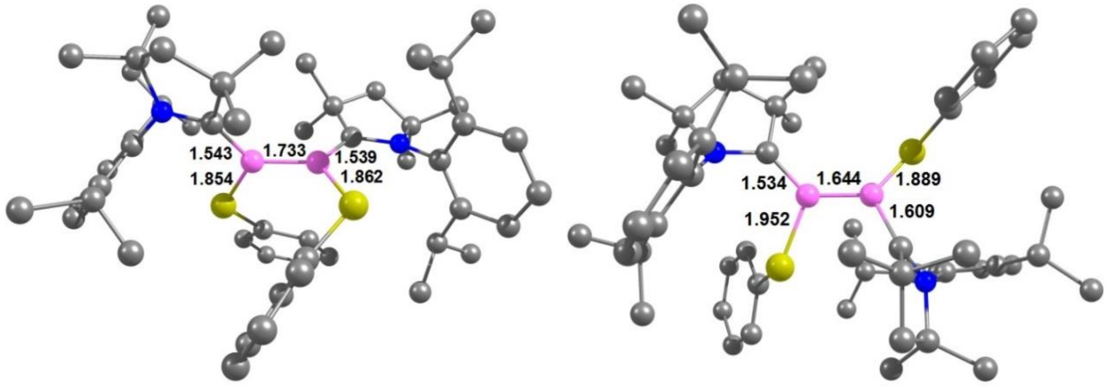

0.0 23.6

T1 S0

Supplementary Figure 26. Optimized geometries of triplet (left) and singlet (right) states of **2b** at the M05-2X/def2-SVP level. The energies are in kcal mol^{-1} and bond lengths are in Å.

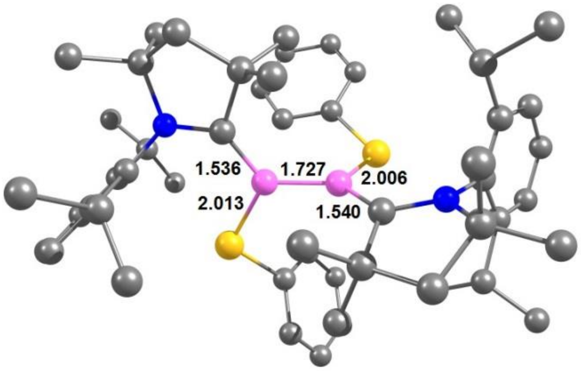

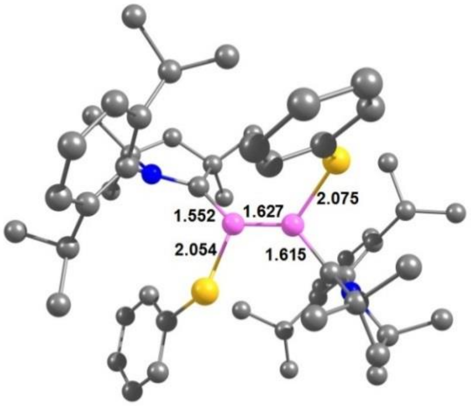

0.0 28.5

T1 S0

Supplementary Figure 27. Optimized geometries of triplet (left) and singlet (right) states of **2c** at the M05-2X/def2-SVP level. The energies are in kcal mol^{-1} and bond lengths are in Å.

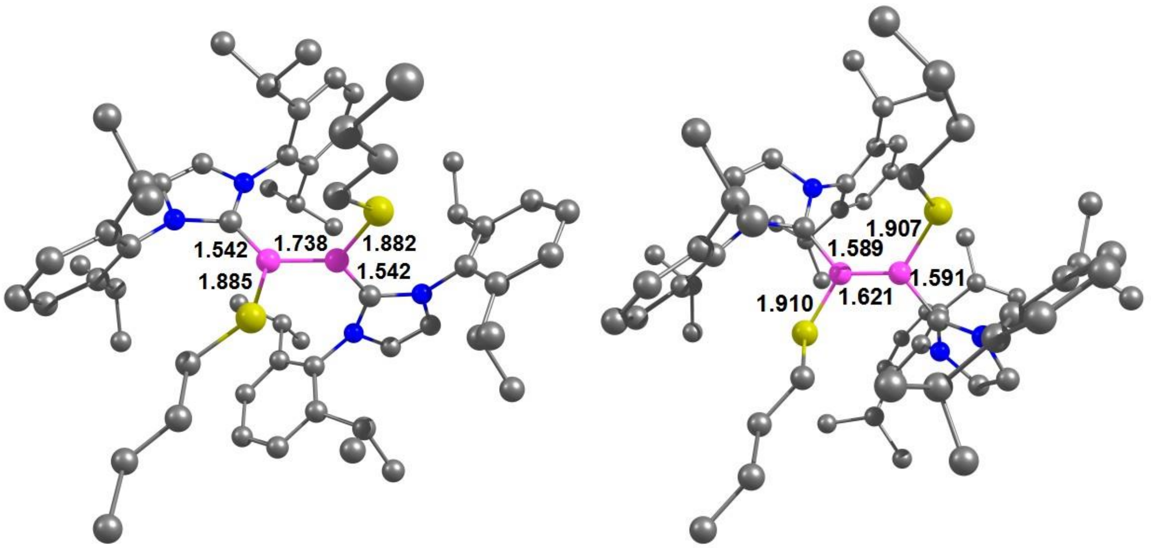

0.0 -17.8

T1 S0

Supplementary Figure 28. Optimized geometries of triplet (left) and singlet (right) states of **1a** at the B3LYP/def2-SVP level. The energies are in kcal mol^{-1} and bond lengths are in Å.

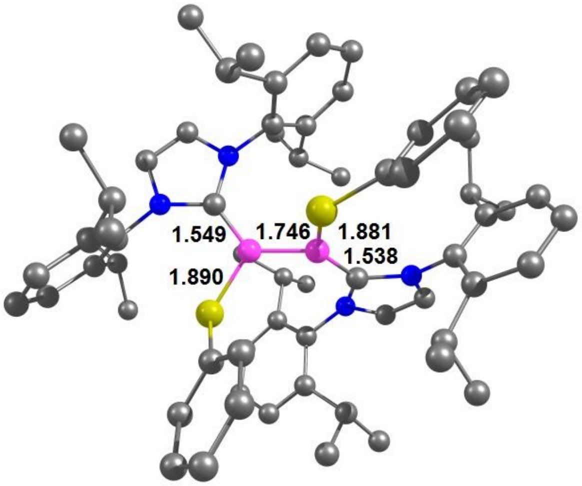

0.0

T1

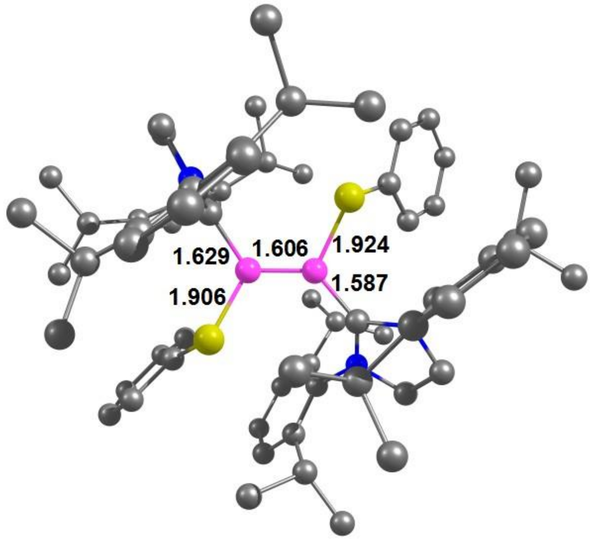

-22.2

S0

Supplementary Figure 29. Optimized geometries of triplet (left) and singlet (right) states of **1b** at the B3LYP/def2-SVP level. The energies are in kcal mol^{-1} and bond lengths are in Å.

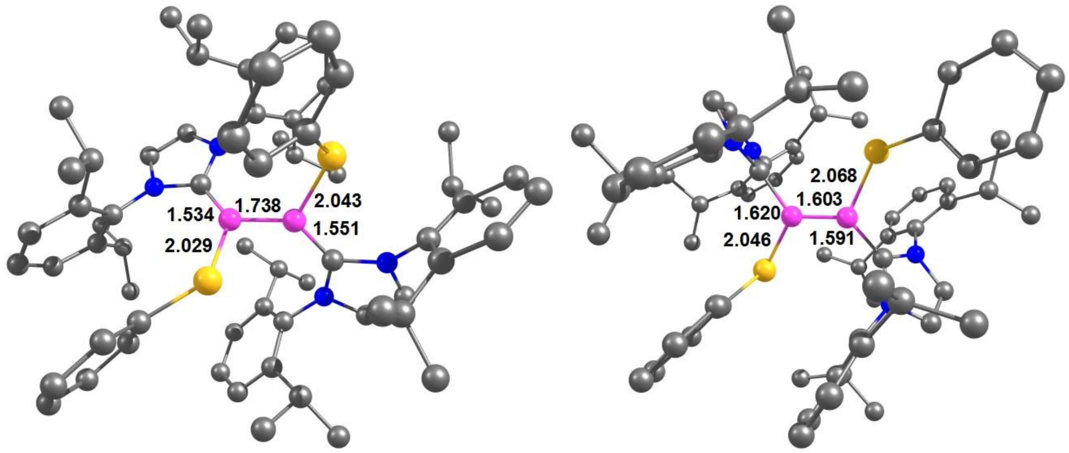

0.0 -23.6

T1 S0

Supplementary Figure 30. Optimized geometries of triplet (left) and singlet (right) states of **1c** at the B3LYP/def2-SVP level. The energies are in kcal mol^{-1} and bond lengths are in Å.

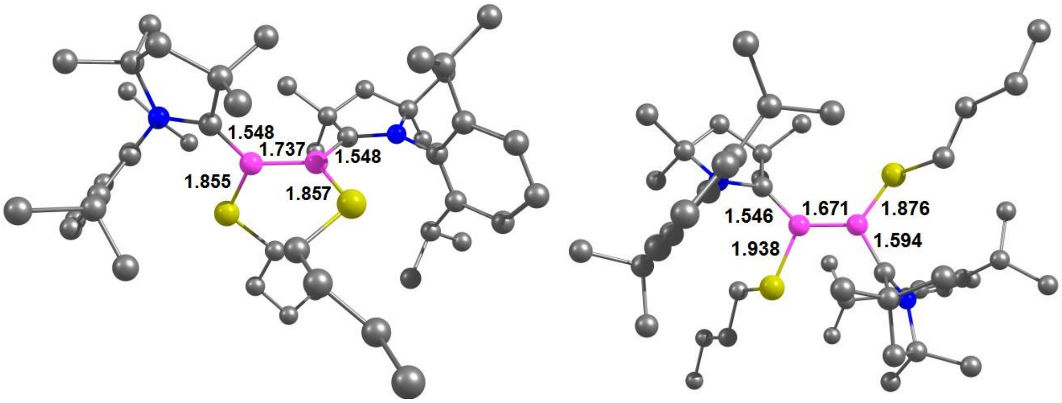

0.0 28.6

T1 S0

Supplementary Figure 31. Optimized geometries of triplet (left) and singlet (right) states of **2a** at the B3LYP/def2-SVP level. The energies are in kcal mol^{-1} and bond lengths are in Å.

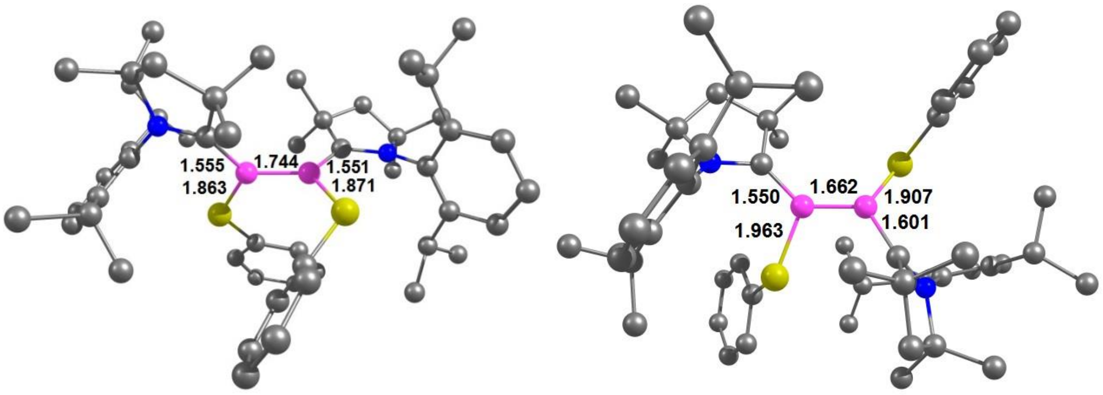

0.0 T1

20.3 S0

Supplementary Figure 32. Optimized geometries of triplet (left) and singlet (right) states of **2b** at the B3LYP/def2-SVP level. The energies are in kcal mol^{-1} and bond lengths are in Å.

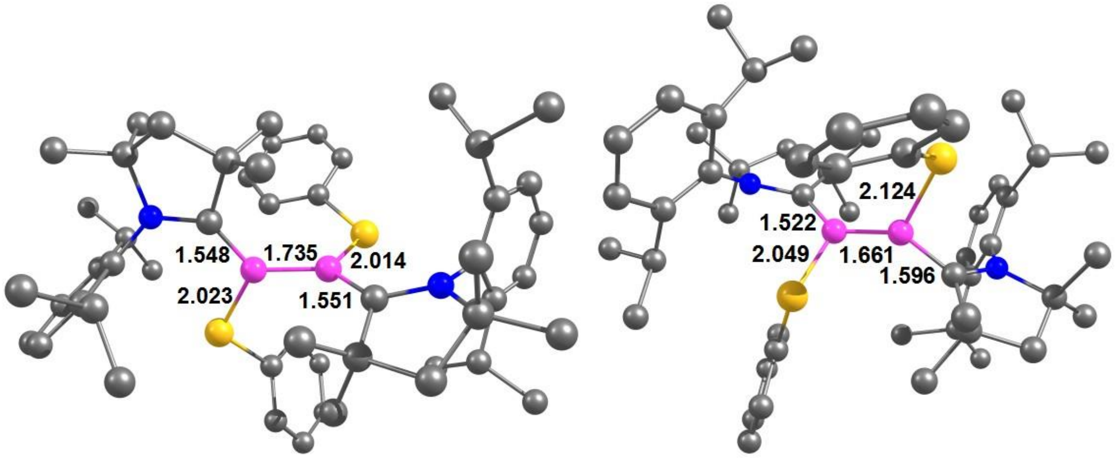

0.0 T1

20.6 S0

Supplementary Figure 33. Optimized geometries of triplet (left) and singlet (right) states of **2c** at the B3LYP/def2-SVP level. The energies are in kcal mol^{-1} and bond lengths are in Å.

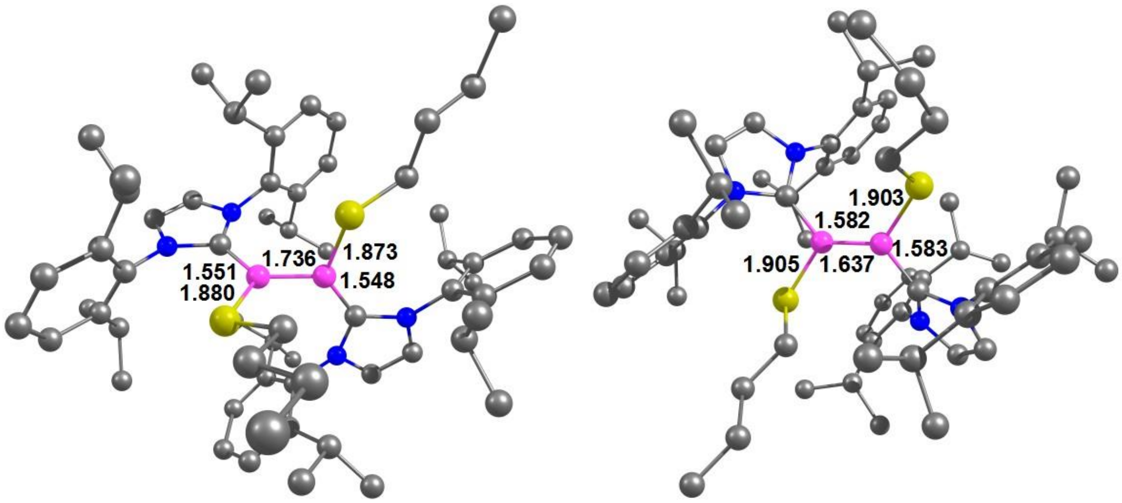

0.0 -19.1

T1 S0

Supplementary Figure 34. Optimized geometries of triplet (left) and singlet (right) states of **1a** at the BP86/def2-SVP level. The energies are in kcal mol^{-1} and bond lengths are in Å.

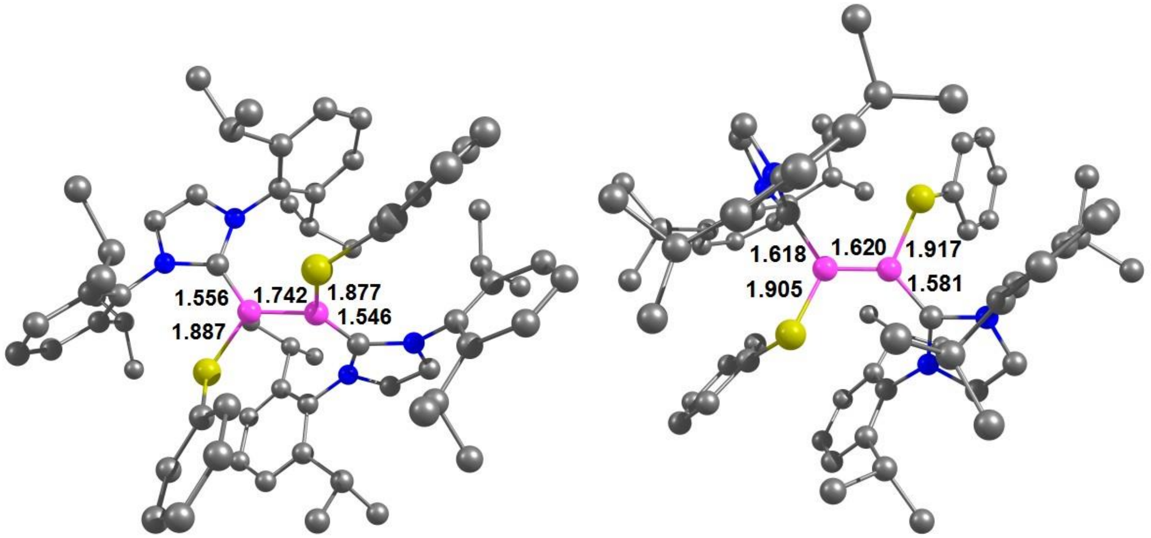

0.0

T1

-21.8

S0

Supplementary Figure 35. Optimized geometries of triplet (left) and singlet (right) states of **1b** at the BP86/def2-SVP level. The energies are in kcal mol^{-1} and bond lengths are in Å.

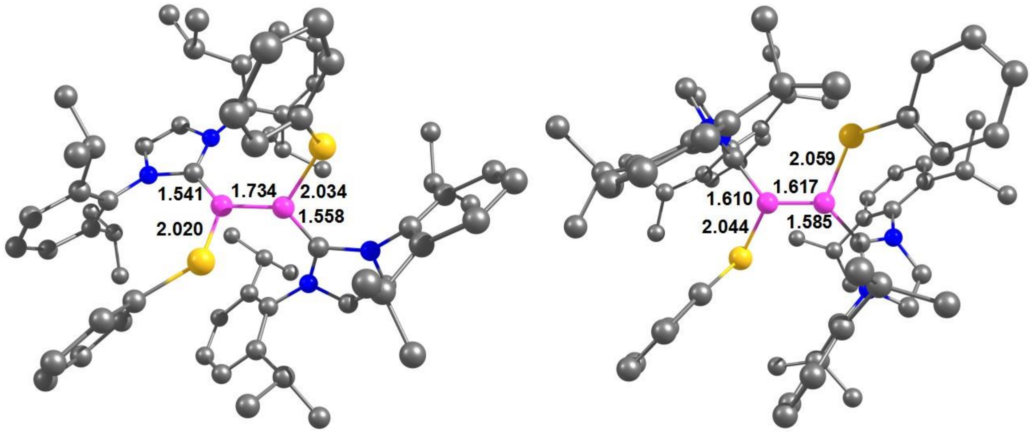

0.0 T1

-23.0 S0

Supplementary Figure 36. Optimized geometries of triplet (left) and singlet (right) states of **1c** at the BP86/def2-SVP level. The energies are in kcal mol^{-1} and bond lengths are in Å.

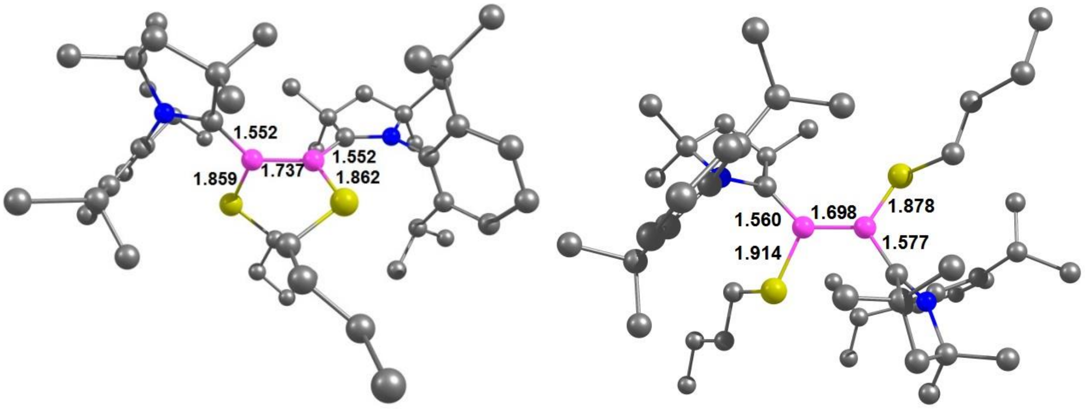

0.0 23.6

T1 S0

Supplementary Figure 37. Optimized geometries of triplet (left) and singlet (right) states of **2a** at the BP86/def2-SVP level. The energies are in kcal mol^{-1} and bond lengths are in Å.

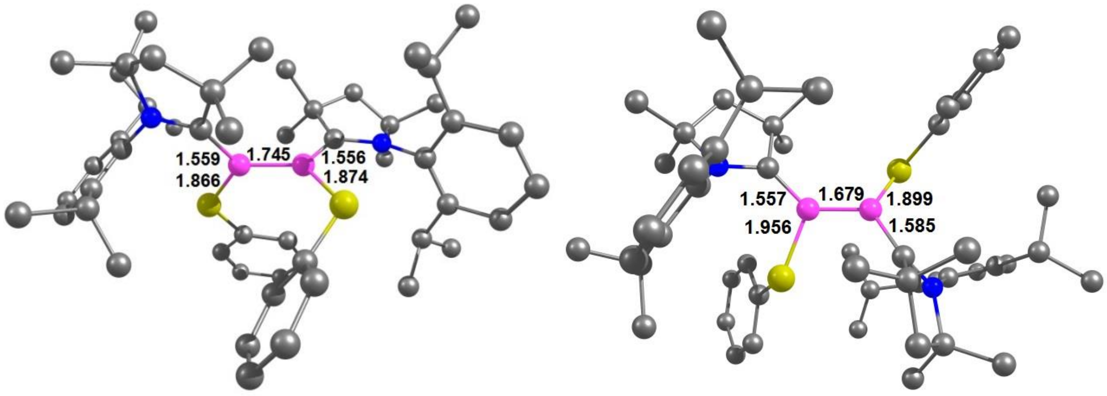

0.0 T1

15.2 S0

Supplementary Figure 38. Optimized geometries of triplet (left) and singlet (right) states of **2b** at the BP86/def2-SVP level. The energies are in kcal mol^{-1} and bond lengths are in Å.

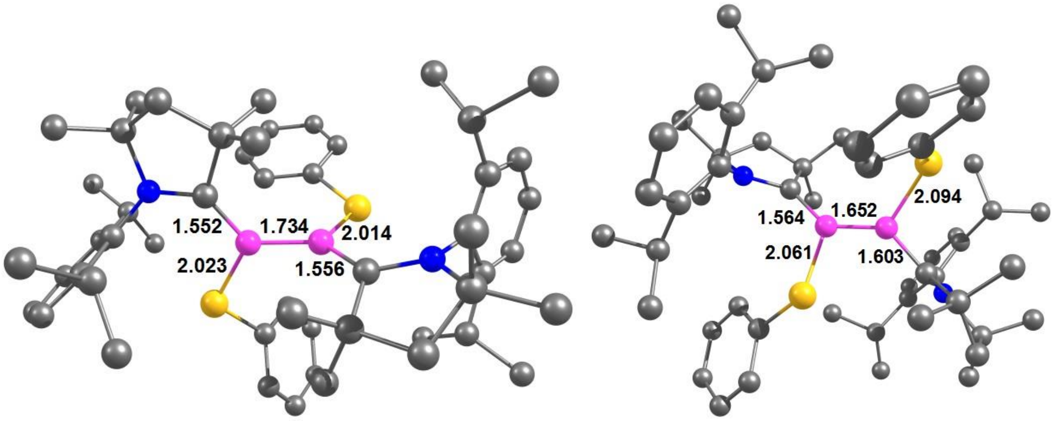

0.0 20.1

T1 S0

Supplementary Figure 39. Optimized geometries of triplet (left) and singlet (right) states of **2c** at the BP86/def2-SVP level. The energies are in kcal mol^{-1} and bond lengths are in Å.

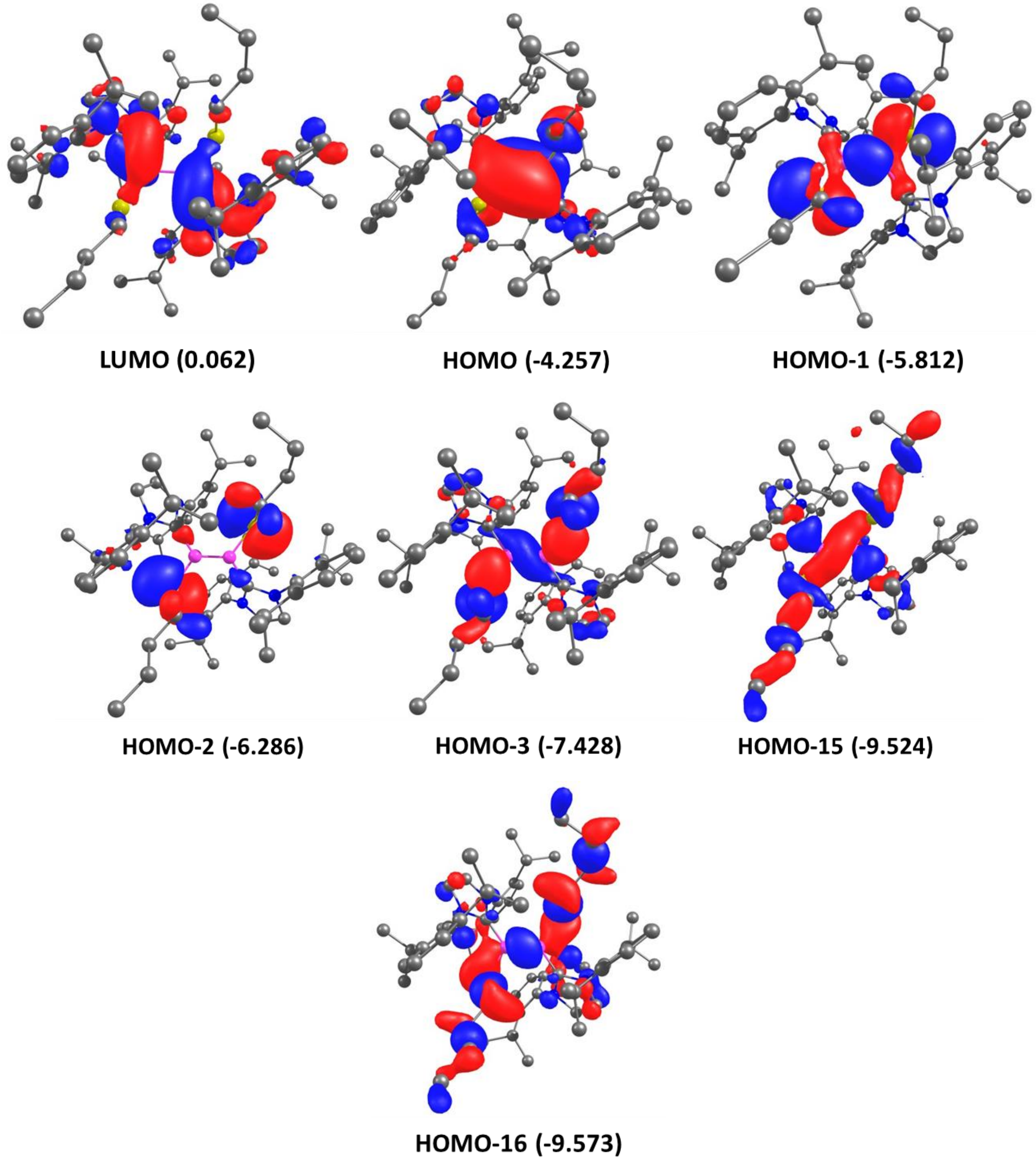

Supplementary Figure 40. Plots of the molecular orbitals (eV) of **1a** at the M05-2X/def2-SVP level.

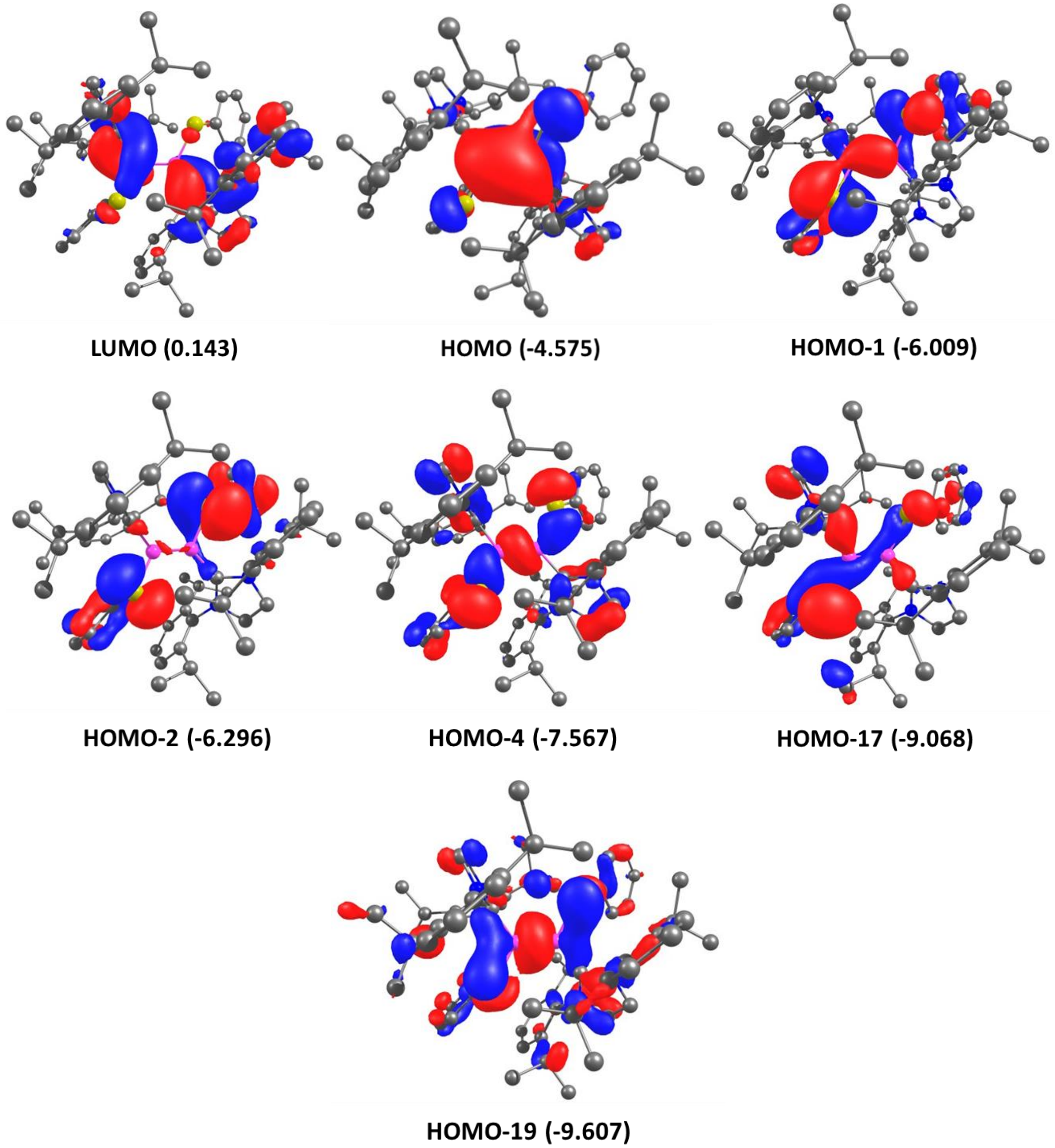

Supplementary Figure 41. Plots of the molecular orbitals (eV) of **1b** at the M05-2X/def2-SVP level.

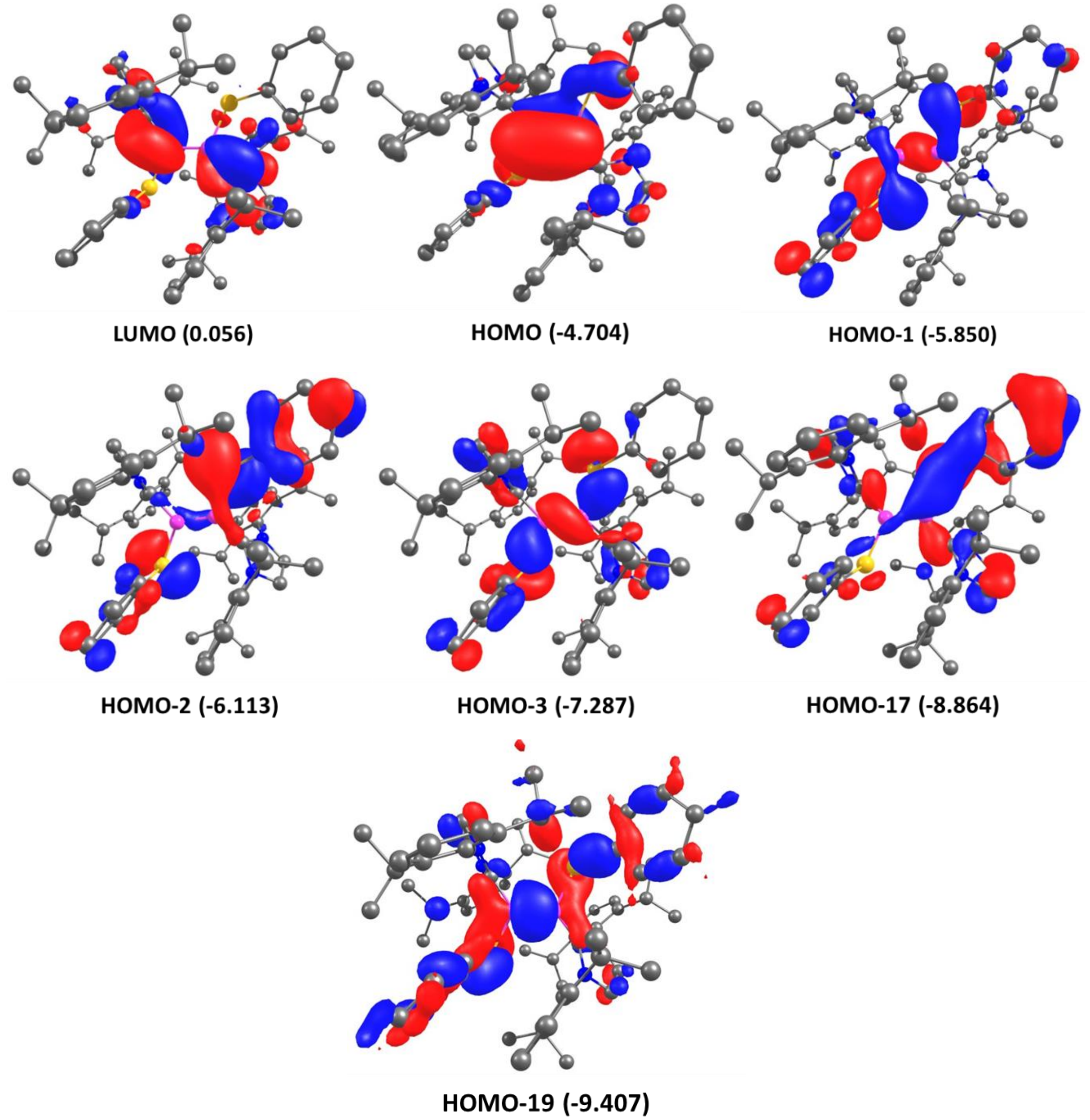

Supplementary Figure 42. Plots of the molecular orbitals (eV) of **1c** at the M05-2X/def2-SVP level.

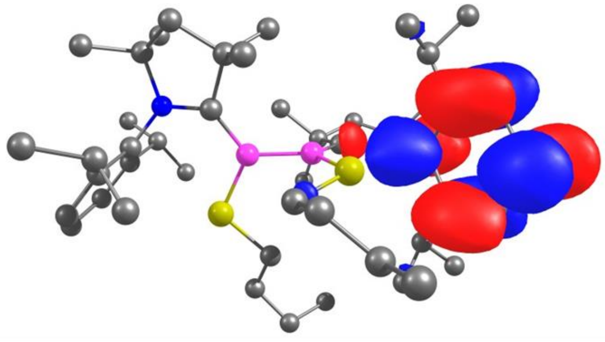

LUMOa (0.810)

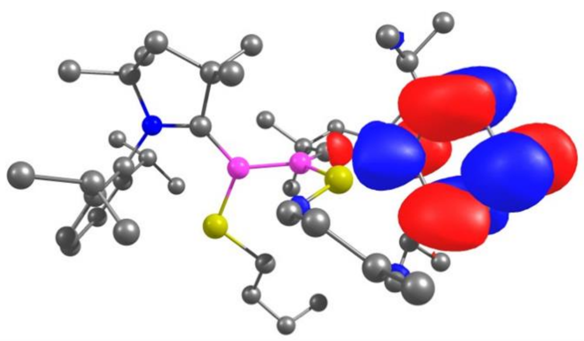

LUMO+2b (0.821)

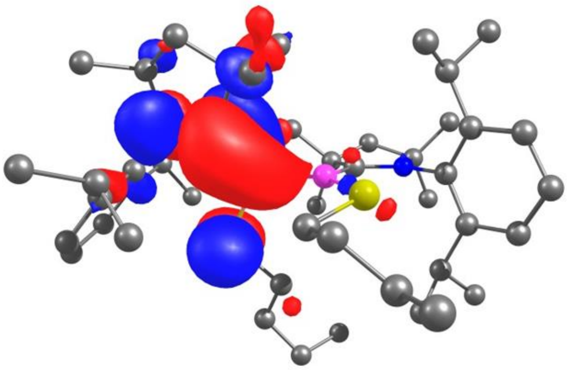

SOMO+1a (-5.113)

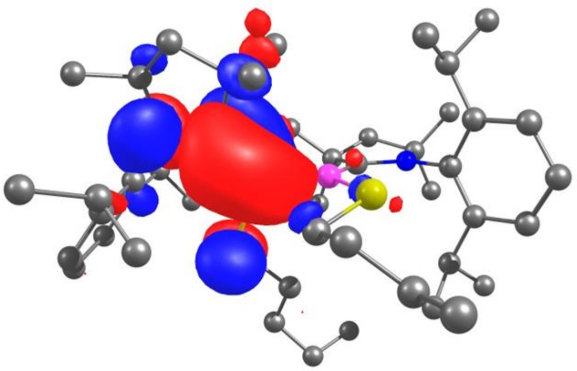

LUMO+1b (-0.425)

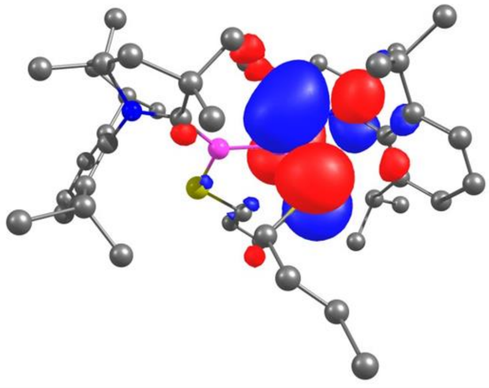

SOMOa (-5.186)

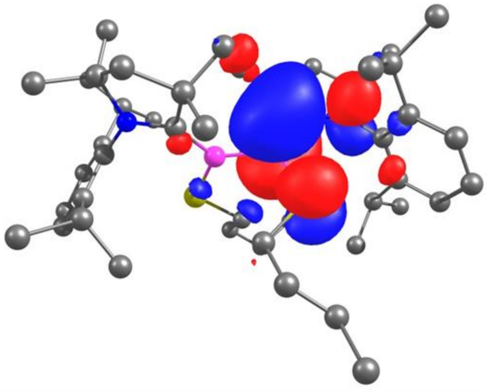

LUMOb (-0.553)

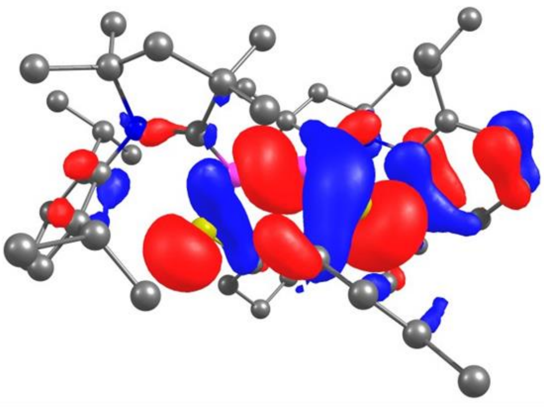

HOMOa (-7.300)

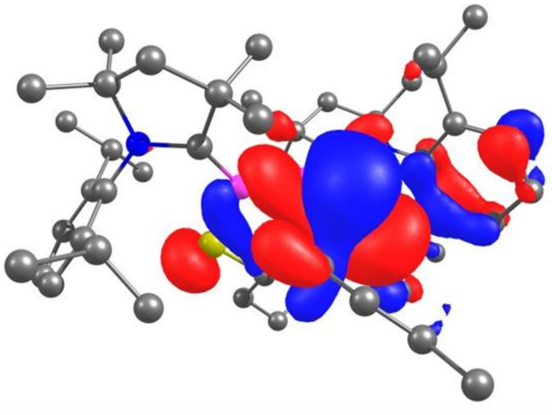

HOMOb (-7.088)

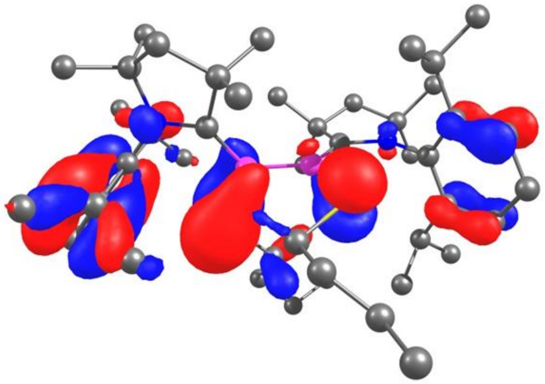

HOMO-1a (-7.546)

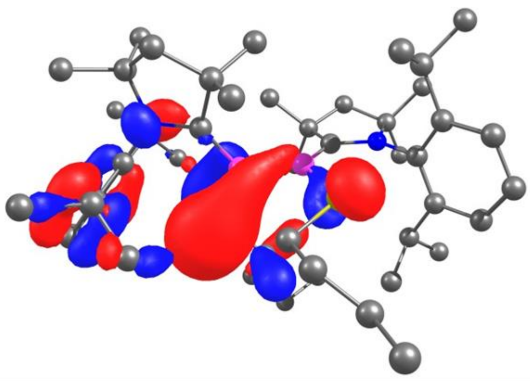

HOMO-1b (-7.277)

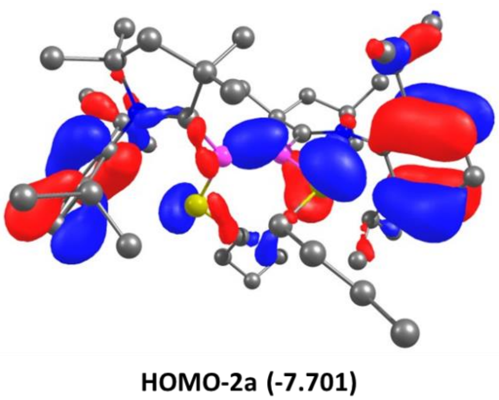

HOMO-2a (-7.701)

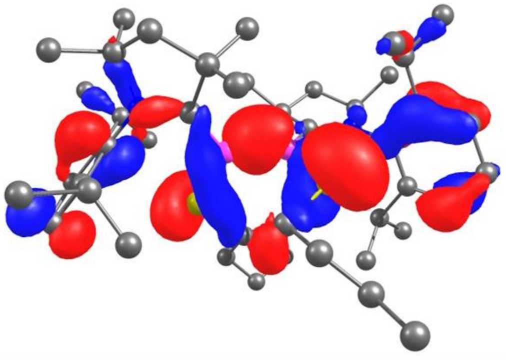

HOMO-2b (-7.575)

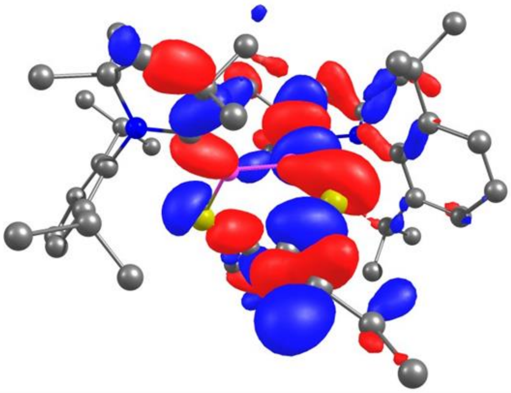

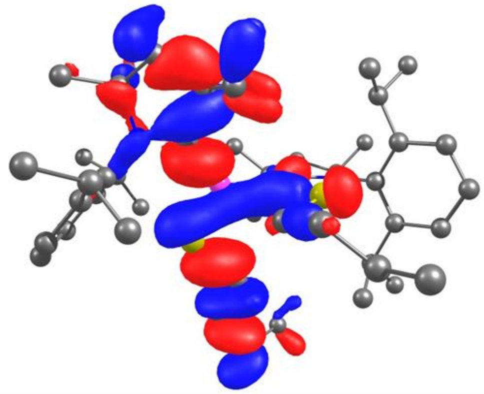

HOMO-10a (-9.656) **HOMO-10b (-9.541)**

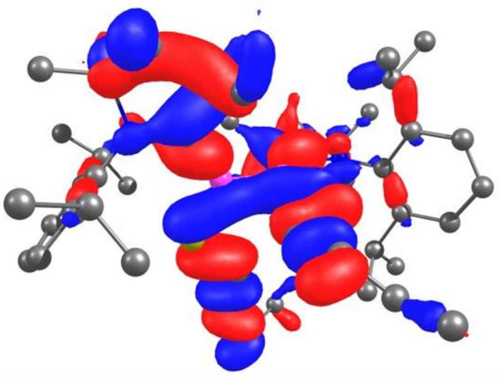

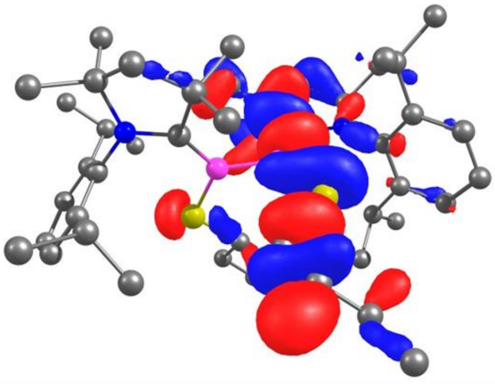

HOMO-11a (-9.711) **HOMO-11b (-9.568)**

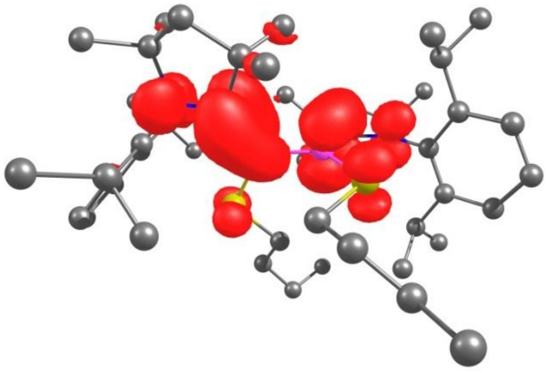

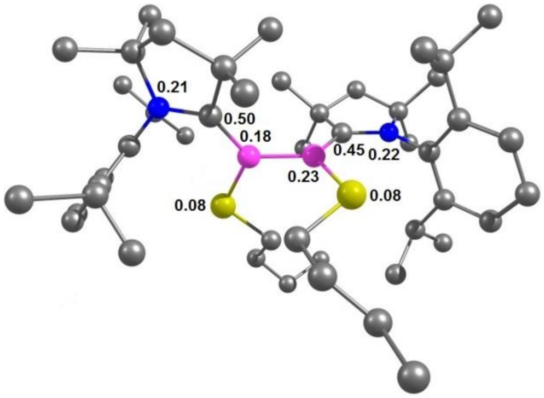

Spin Density **Mulliken spin densities**

Supplementary Figure 43. Plots of the molecular orbitals (eV), spin density and Mulliken spin densities of **2a** at the M05-2X/def2-SVP level.

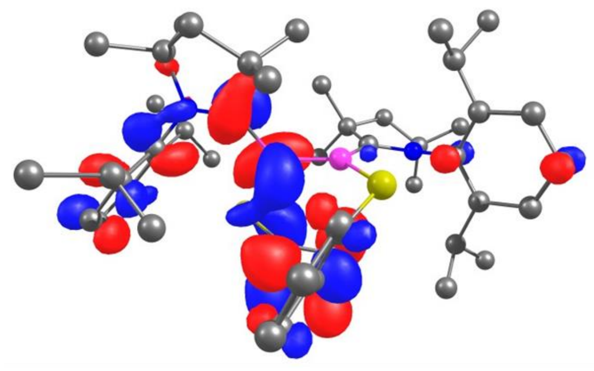

LUMOa (0.663)

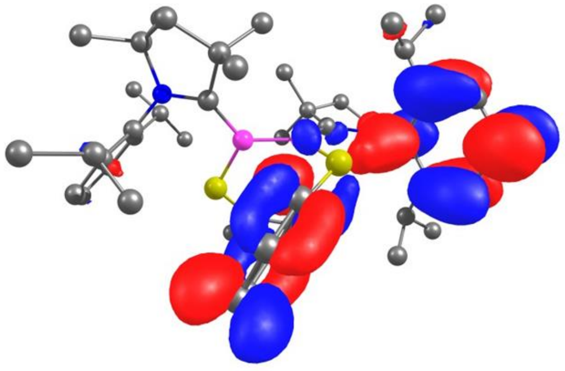

LUMO+2b (0.747)

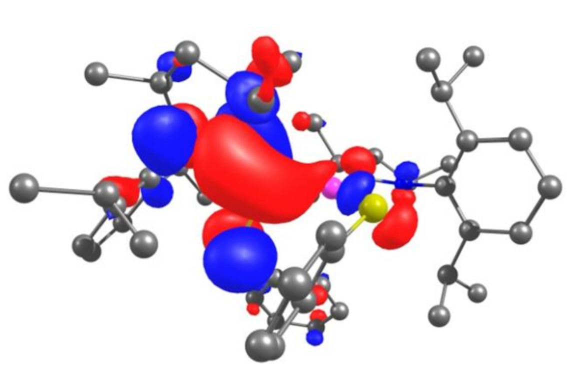

SOMO+1a (-5.203)

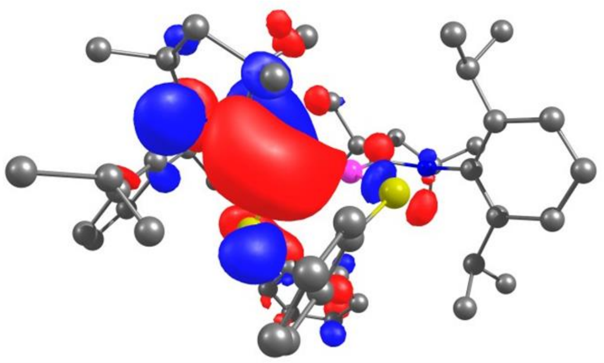

LUMO+1b (-0.706)

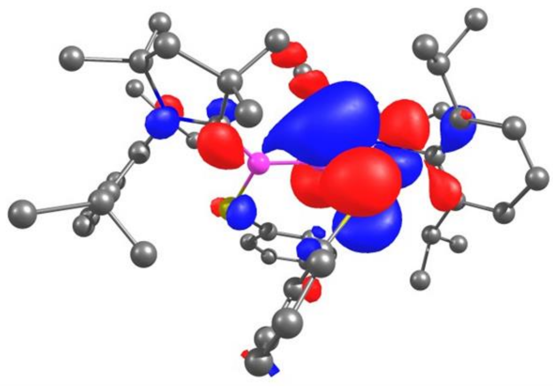

SOMOa (-5.360)

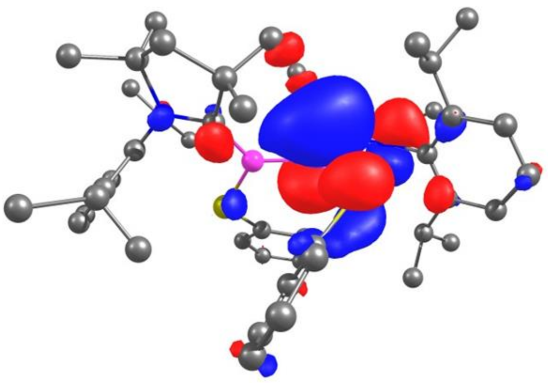

LUMOb (-0.813)

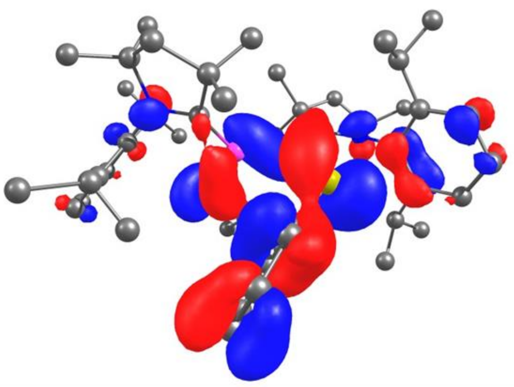

HOMOa (-7.226)

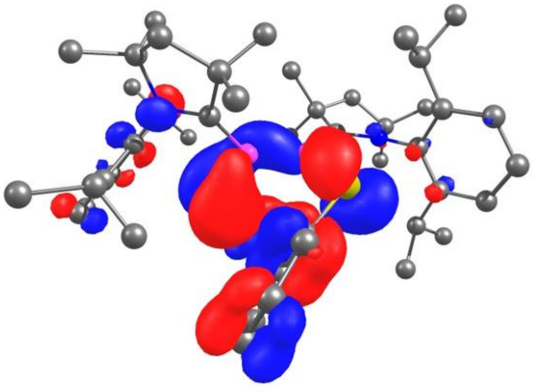

HOMOb (-6.933)

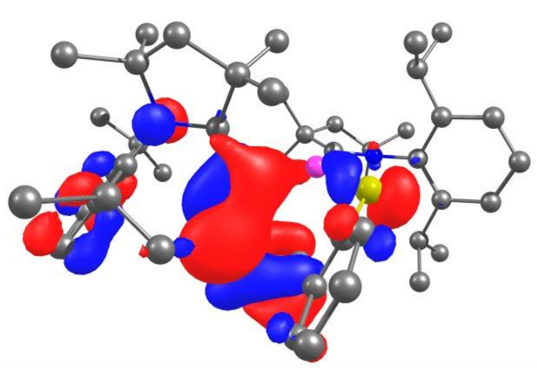

HOMO-1a (-7.300)

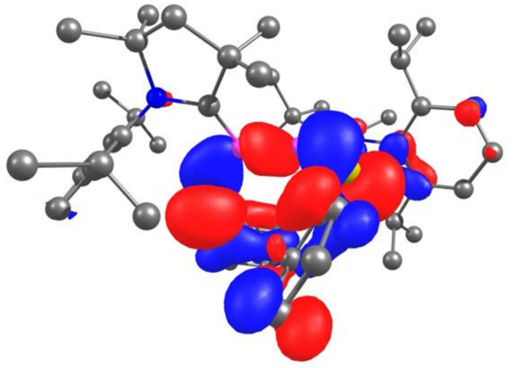

HOMO-1b (-7.088)

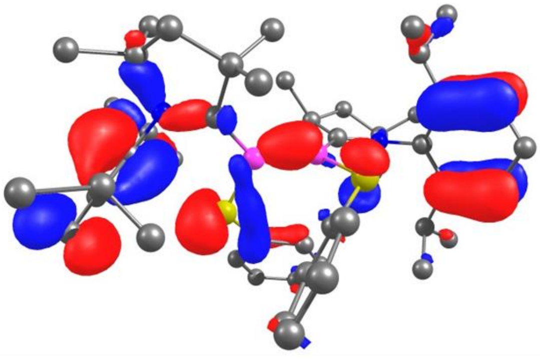

HOMO-2a (-7.766)

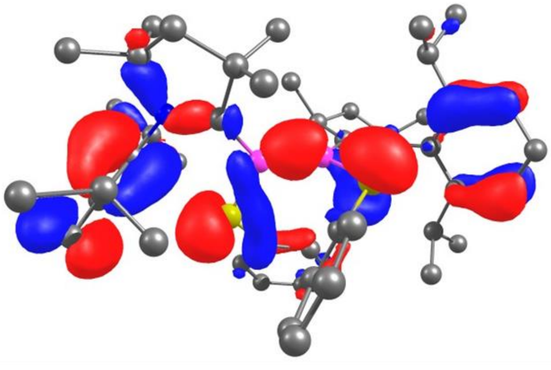

HOMO-2b (-7.701)

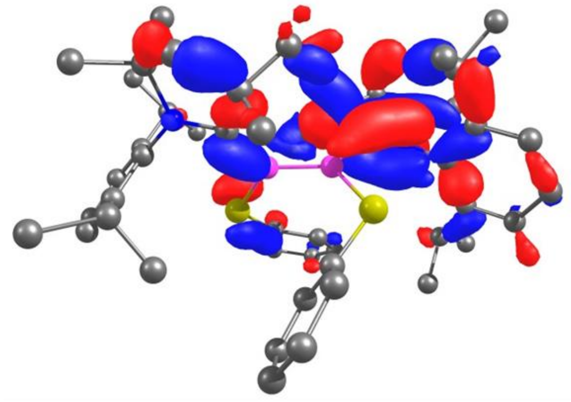

HOMO-12a (-9.409)

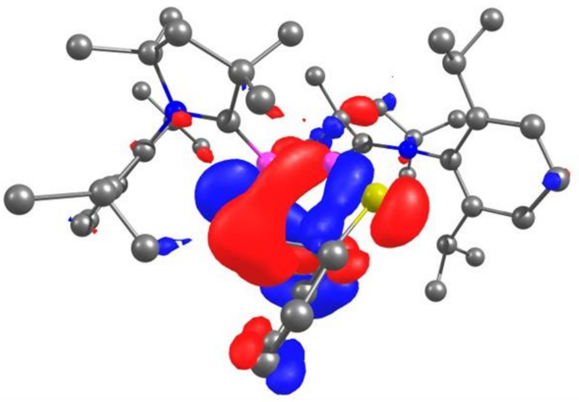

HOMO-12b (-9.133)

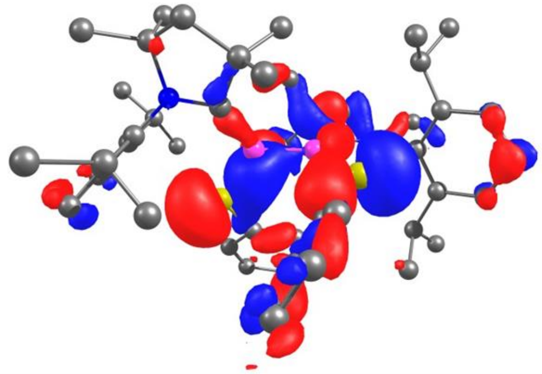

HOMO-13a (-9.713)

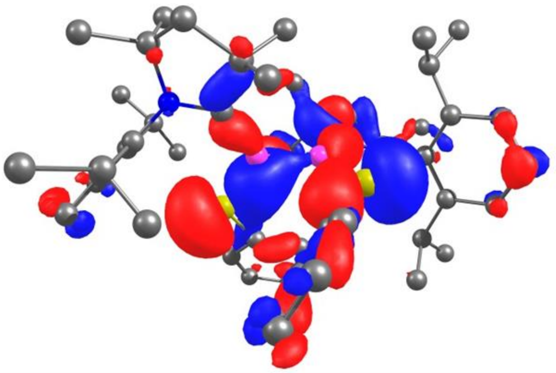

HOMO-13b (-9.646)

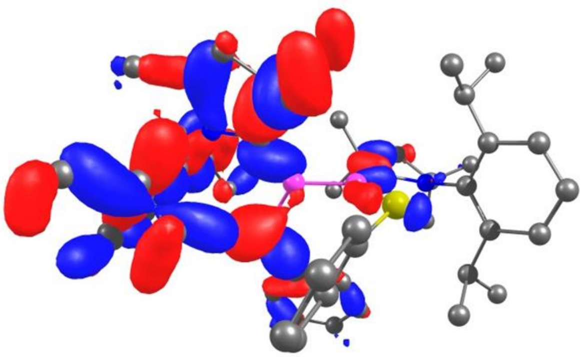

HOMO-16a (-10.064)

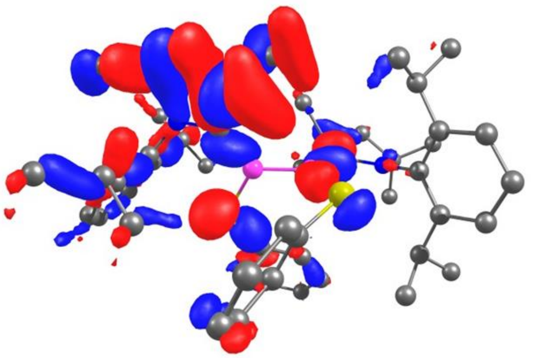

HOMO-16b (-9.983)

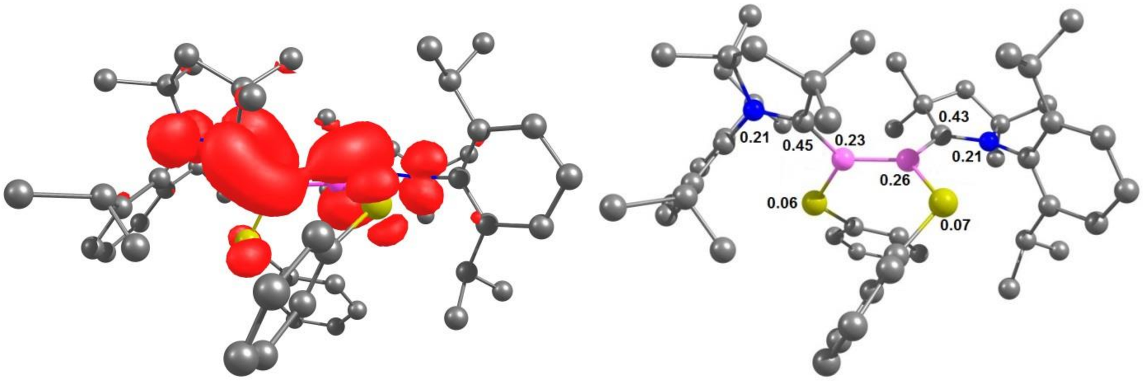

Spin Density **Mulliken spin densities**

Supplementary Figure 44. Plots of the molecular orbitals (eV), spin density and Mulliken spin densities of **2b** at the M05-2X/def2-SVP level.

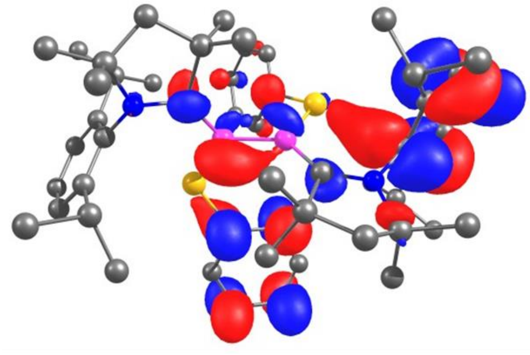

LUMOa (0.640)

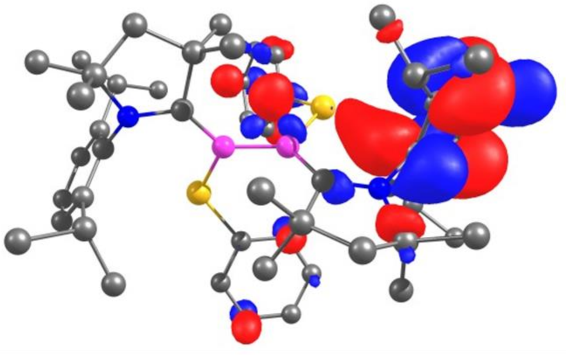

LUMO+2b (0.708)

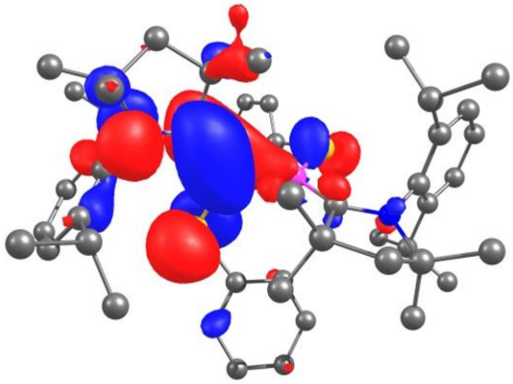

SOMO+1a (-5.221)

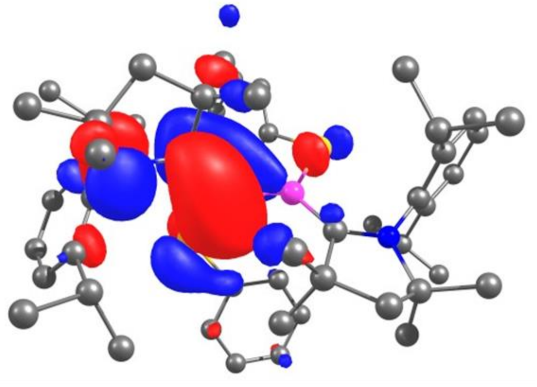

LUMO+1b (-0.781)

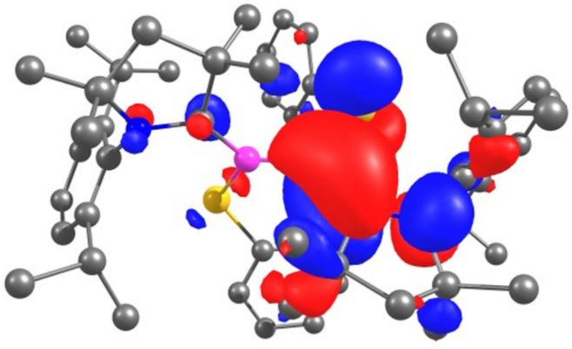

SOMOa (-5.399)

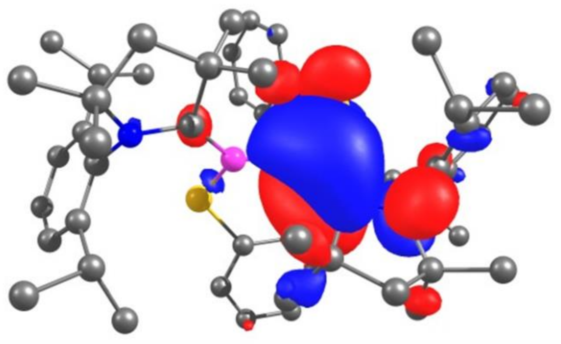

LUMOb (-0.932)

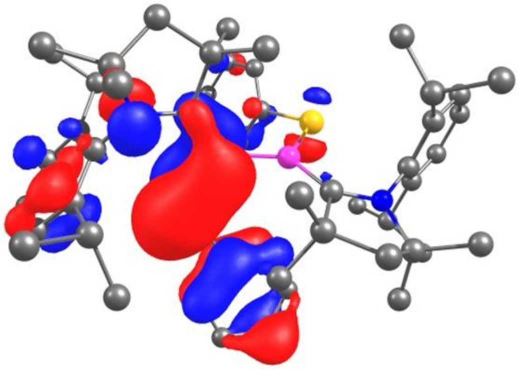

HOMOa (-7.061)

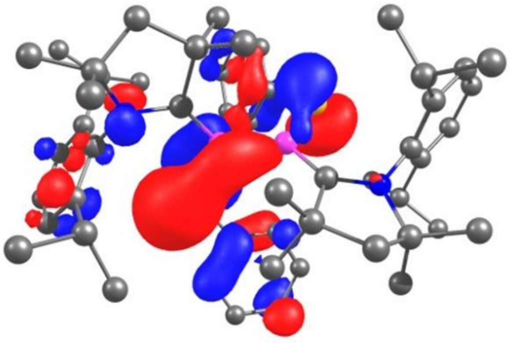

HOMOb (-6.722)

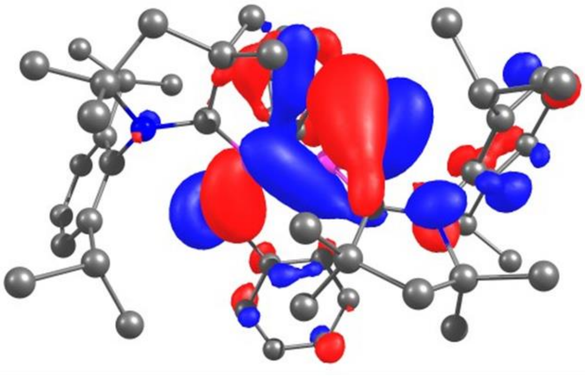

HOMO-1a (-7.101)

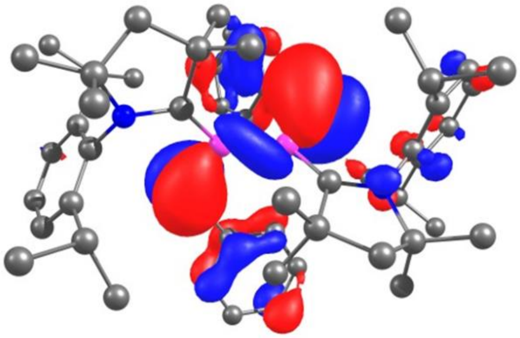

HOMO-1b (-6.902)

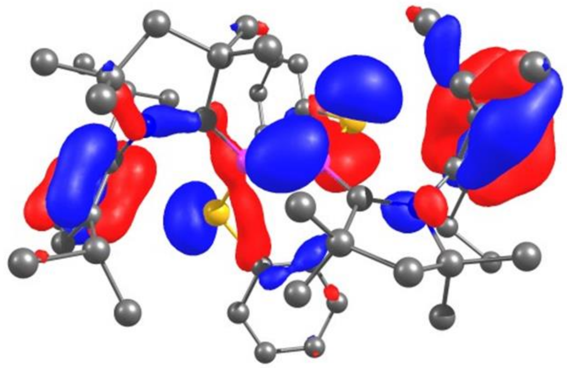

HOMO-2a (-7.721)

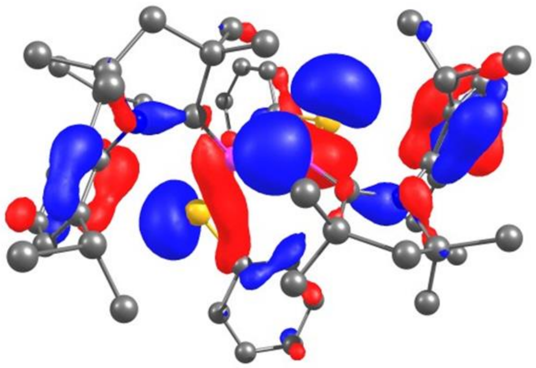

HOMO-2b (-7.589)

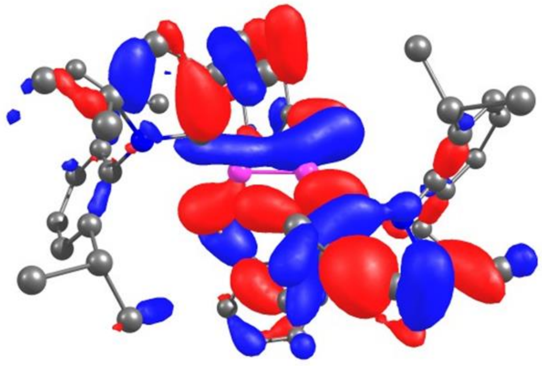

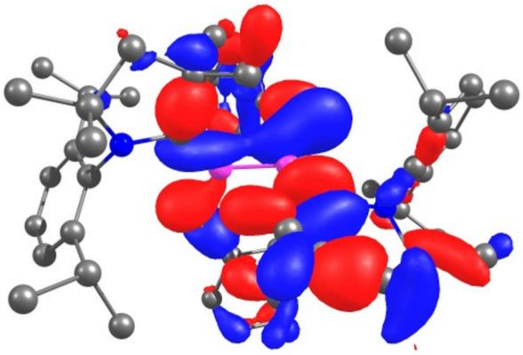

HOMO-14a (-9.872) **HOMO-14b (-9.736)**

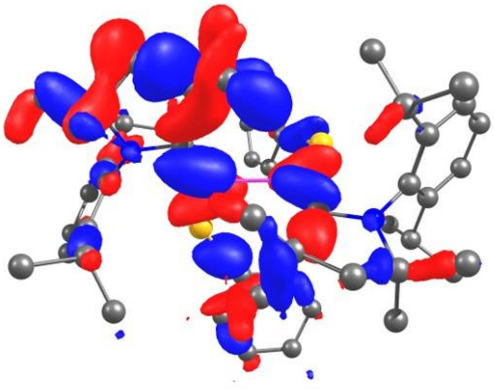

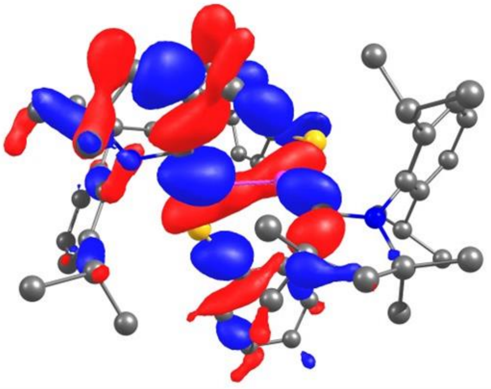

HOMO-15a (-9.903) **HOMO-15b (-9.768)**

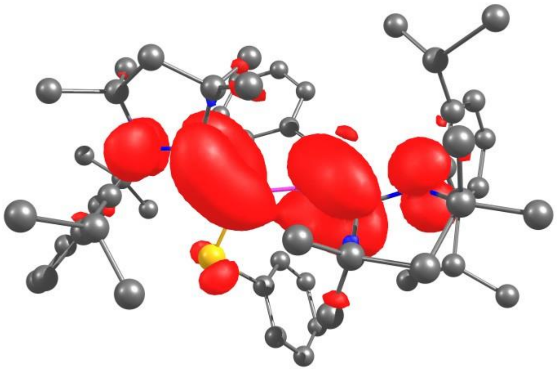

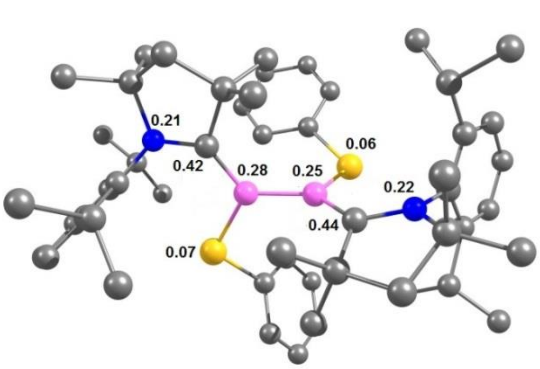

Spin Density **Mulliken spin densities**

Supplementary Figure 45. Plots of the molecular orbitals (eV), spin density and Mulliken spin densities of **2c** at the M05-2X/def2-SVP level.

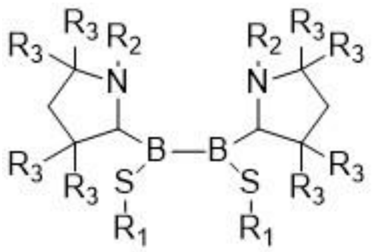

Supplementary Figure 46. To achieve the model compound **2a’** the following substituents were changed: R_1: *n*Bu to Me, R_2: Dip to Me, and R_3: Me to H.

Supplementary tables

Supplementary Table 1. Relative energies (kcal mol^{-1}) calculated at the M05-2X/def2-SVP, B3LYP/def2-SVP and BP86/def2-SVP levels.

	M0-2X/def2-SVP	B3LYP/def2-SVP	BP86/def2-SVP
**1a** (T1)	0.0	0.0	0.0
**1a** (S0)	-16.7	-17.8	-19.1
**1b** (T1)	0.0	0.0	0.0
**1b** (S0)	-24.4	-22.2	-21.8
**1c** (T1)	0.0	0.0	0.0
**1c** (S0)	-26.3	-23.6	-23.0
**2a** (T1)	0.0	0.0	0.0
**2a** (S0)	33.2	28.6	23.6
**2b** (T1)	0.0	0.0	0.0
**2b** (S0)	23.6	20.3	15.2
**2c** (T1)	0.0	0.0	0.0
**2c** (S0)	28.5	20.6	20.1

Supplementary Table 2. NBO results for **1a-c** and **2a-c** at the M05-2X/def2-SVP level. Partial charges are given in electrons.

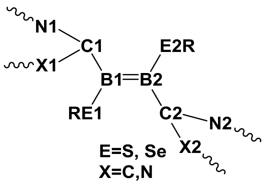

Charges

	q(C1)	q(C2)	q(B1)	q(B2)	q(E1)	q(E2)	q(N1)	q(N2)	q(X1)	q(X2)
**1a (ER=SBu)**	0.289	0.294	-0.157	-0.156	-0.129	-0.129	-0.419	-0.419	-0.428	-0.428
**1b (ER=SPh)**	0.330	0.283	-0.143	-0.217	-0.012	-0.005	-0.415	-0.426	-0.413	-0.429
**1c (ER=SePh)**	0.323	0.281	-0.212	-0.262	0.086	0.089	-0.415	-0.426	-0.413	-0.429
**2a (ER=SBu)**	-0.110	-0.095	0.161	0.141	-0.004	0.018	-0.479	-0.467	-0.095	-0.092
**2b (ER=SPh)**	-0.094	-0.093	0.164	0.150	0.066	0.040	-0.473	-0.467	-0.095	-0.095
**2c (ER=SePh)**	-0.097	-0.102	0.096	0.107	0.147	0.173	-0.464	-0.470	-0.094	-0.094

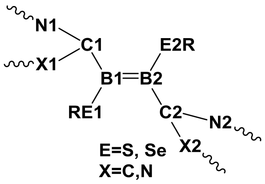

Wiberg bond indices

	B1-B2	B1-C1	B2-C2	B1-E1	B2-E2
**1a (ER=SBu)**	1.550	0.918	0.909	0.946	0.946
**1b (ER=SPh)**	1.578	0.849	0.947	0.951	0.935
**1c (ER=SePh)**	1.602	0.858	0.937	0.966	0.953
**2a (ER=SBu)**	0.974	1.043	1.054	1.180	1.160
**2b (ER=SPh)**	0.976	1.059	1.118	1.130	1.118
**2c (ER=SePh)**	0.990	1.056	1.062	1.120	1.133

Supplementary Table 3. Summary of the single-point computations of the S-T gap performed for model compound **2a'**. The calculations were performed for the geometries obtained from the MN12L functionals as described above. The calculations were performed with the GAUSSIAN16 program suite[1], the ORCA package[2], and Turbomole 7.0[3].

method	S-T gap	Compared to NEVPT2
NEVPT2[4-7]	–0.40	0.00
SCS-MP2[8-10]	–22.88	–22.48
BB1K[11]	–4.77	–4.37
M06L[12]	–11.82	–11.42
M11L[13]	–32.33	–31.93
M062X[14]	–25.98	–25.58
M15L[15]	–11.82	–11.42
MN12L[16]	–6.85	–6.46
MPW1K[17]	–24.38	–23.98
N12SX[18]	–17.54	–17.14
PBE1W[19]	–7.61	–7.22
SOGGA11X[20]	–7.07	–6.67
wB97XD[21,22]	–28.05	–27.65

Supplementary references

1. Gaussian 16, Revision A.03, Frisch, M. J., Trucks, G. W., Schlegel, H. B., Scuseria, G. E., Robb, M. A., Cheeseman, J. R., Scalmani, G., Barone, V., Petersson, G. A., Nakatsuji, H., Li, X., Caricato, M., Marenich, A. V., Bloino, J., Janesko, B. G., Gomperts, R., Mennucci, B., Hratchian, H. P., Ortiz, J. V., Izmaylov, A. F., Sonnenberg, J. L., Williams-Young, D., Ding, F., Lipparini, F., Egidi, F., Goings, J., Peng, B., Petrone, A., Henderson, T., Ranasinghe, D., Zakrzewski, V. G., Gao, J., Rega, N., Zheng, G., Liang, W., Hada, M., Ehara, M., Toyota, K., Fukuda, R., Hasegawa, J., Ishida, M., Nakajima, T., Honda, Y., Kitao, O., Nakai, H., Vreven, T., Throssell, K., Montgomery, Jr., J. A., Peralta, J. E., Ogliaro, F., Bearpark, M. J., Heyd, J. J., Brothers, E. N., Kudin, K. N., Staroverov, V. N., Keith, T. A., Kobayashi, R., Normand, J., Raghavachari, K., Rendell, A. P., Burant, J. C., Iyengar, S. S., Tomasi, J., Cossi, M., Millam, J. M., Klene, M., Adamo, C., Cammi, R., Ochterski, J. W., Martin, R. L., Morokuma, K., Farkas, O., Foresman, J. B., Fox, D. J. Gaussian, Inc., Wallingford CT, 2016
2. Neese, F., Software update: the ORCA program system, version 4.0. *WIREs Comput. Mol. Sci.*, **7**, e1327 (2017).
3. TURBOMOLE V7.0 2015, a development of University of Karlsruhe and Forschungszentrum Karlsruhe GmbH, 1989-2007, TURBOMOLE GmbH, since 2007; available from http://www.turbomole.com.
4. Angeli, C., Borini, S., Cimiraglia, R. An application of second-order n-electron valence state perturbation theory to the calculation of excited states. *Theor. Chem. Acc.* **111**, 352 (2004).
5. Angeli, C., Cimiraglia, R., Malrieu, J. P. n-electron valence state perturbation theory: A spinless formulation and an efficient implementation of the strongly contracted and of the partially contracted variants. *J. Chem. Phys.* **117**, 9138 (2002).
6. Angeli, C., Cimiraglia, R., Evangelisti, S., Leininger, T., Malrieu, J. P. Introduction of n-electron valence states for multireference perturbation theory. *J. Chem. Phys.* **114**, 10252-10264 (2001).
7. Angeli, C., Cimiraglia, R., Malrieu, J. P. N-electron valence state perturbation theory: a fast implementation of the strongly contracted variant. *Chem. Phys. Lett.* **350**, 297-305 (2001).

8. Hättig, C., Weigend, F. CC2 Excitation Energy Calculations on Large Molecules Using the Resolution of the Identity Approximation. *J. Chem. Phys.* **113**, 5154−5161 (2000).
9. Hättig, C., Hellweg, A., Köhn, A. Distributed Memory Parallel Implementation of Energies and Gradients for Second-Order Møller-Plesset Perturbation Theory with the Resolution-of-the-Identity Approximation. *Phys. Chem. Chem. Phys.* **8**, 1159−1169 (2006).
10. Grimme, S. Improved Second-Order Møller−Plesset Perturba-tion Theory by Separate Scaling of Parallel- and Antiparallel-Spin Pair Correlation Energies. *J. Chem. Phys.* **118**, 9095−9102 (2003).
11. Zhang, Y., Yang, W. Comment on "Generalized Gradient Approximation Made Simple". *Phys. Rev. Lett.* **80**, 890 (1998).
12. Zhao, Y., Truhlar, D. G. A new local density functional for main-group thermochemistry, transition metal bonding, thermochemical kinetics, and noncovalent interactions. *J. Chem. Phys.* **125**, 194101-194118 (2006).
13. Peverati, R., Truhlar, D. G. M11-L: A Local Density Functional That Provides Improved Accuracy for Electronic Structure Calculations in Chemistry and Physics. *J. Phys. Chem. Lett.* **3**, 117-124 (2012).
14. Zhao Y., Truhlar, D. G. The M06 suite of density functionals for main group thermochemistry, thermochemical kinetics, noncovalent interactions, excited states, and transition elements: two new functionals and systematic testing of four M06-class functionals and 12 other functionals. *Theor. Chem. Acc.* **120**, 215-241 (2008).
15. Yu, H. S., He, X., Truhlar, D. G. MN15-L: A New Local Exchange-Correlation Functional for Kohn–Sham Density Functional Theory with Broad Accuracy for Atoms, Molecules, and Solids. *J. Chem. Theor. Comput.* **12**, 1280-1293 (2016).
16. Peverati R., Truhlar, D. G. An improved and broadly accurate local approximation to the exchange–correlation density functional: The MN12-L functional for electronic structure calculations in chemistry and physics. *Phys. Chem. Chem. Phys.* **10**, 13171 (2012).
17. Lynch, B. J., Fast, P. L., Harris, M., Truhlar, D. G. Adiabatic Connection for Kinetics. *J. Phys. Chem. A* **104**, 4811 (2000).
18. Peverati R., Truhlar, D. G., Exchange-Correlation Functional with Good Accuracy for Both Structural and Energetic Properties while Depending Only on the Density and Its Gradient. *J. Chem. Theor. Comput.* **8** (2012) 2310-2319.

19. Dahlke, E. E., Truhlar, D. G. Improved Density Functionals for Water. *J. Phys. Chem. B* **109**, 15677-15683 (2005).

20. Peverati R., Truhlar, D. G. A global hybrid generalized gradient approximation to the exchange-correlation functional that satisfies the second-order density-gradient constraint and has broad applicability in chemistry. *J. Chem. Phys.* **135**, 191102-191104 (2011).

21. Chai, J.-D., Head-Gordon, M. Systematic optimization of long-range corrected hybrid density functionals, *J. Chem. Phys.* **128**, 084106 (2008).

22. Chai, J.-D., Head-Gordon, M. Long-range corrected hybrid density functionals with damped atom-atom dispersion corrections, *Phys. Chem. Chem. Phys.* **10**, 6615-6620 (2008).
