# Supplementary material for: Isolation of diborenes and their 90°-twisted diradical congeners
Source: Nat Commun. 2018 Mar 22;9:1197. doi: 10.1038/s41467-018-02998-3 (PMC5864745; doi:10.1038/s41467-018-02998-3)
Supplement: Supplementary file 3 — Description of Additional Supplementary Files(PDF 166 kb) [file 41467_2018_2998_MOESM3_ESM.pdf]

Description of Additional Supplementary Files

File Name: Supplementary Data 1
Description: Cartesian coordinates (Å) and energy (a.u) of the calculated compounds.
